# The antimicrobial peptide Cec4 has therapeutic potential against clinical carbapenem-resistant *Klebsiella pneumoniae*

**DOI:** 10.1128/spectrum.02738-24

**Published:** 2025-05-16

**Authors:** Lu Li, Yang Zeng, Minfang Tian, Huijun Cao, Zhilang Qiu, Guo Guo, Feng Shen, Yuping Wang, Jian Peng

**Affiliations:** 1 Department of Intensive Care Unit/Centre for Clinical Laboratories, Affiliated Hospital of Guizhou Medical University 74720, Guiyang, Guizhou, People's Republic of China; 2 Key Laboratory of Infectious Immune and Antibody Engineering of Guizhou Province, Cellular Immunotherapy Engineering Research Center of Guizhou Province, School of Biology and Engineering/School of Basic Medical Sciences, Guizhou Medical University 74628, Guiyang, Guizhou, People's Republic of China; 3 Guizhou Institute of Precision Medicine, Affiliated Hospital of Guizhou Medical University 74720, Guiyang, Guizhou, People's Republic of China; 4 The Engineering Research Center of Health Medicine Biotechnology of Institution of Higher Education of Guizhou Province, Guiyang, Guizhou, People's Republic of China; Duke University, Durham, North Carolina, USA

**Keywords:** *Klebsiella pneumoniae*, antibacterial activity, antibacterial mechanism, antimicrobial peptide, CRKP

## Abstract

The rapid increase in carbapenem-resistant *Klebsiella pneumoniae* (CRKP) infections, along with the cross-resistance of CRKP to other antibiotics, has created an urgent need for novel therapeutic agents. Among the potential options for next-generation antibiotics, antimicrobial peptides (AMPs) show great promise. In this study, we aimed to elucidate the mechanisms underlying the antibacterial activity against CRKP of an antibacterial peptide named Cecropin-4 (Cec4), which we successfully identified previously. Our results demonstrate that Cec4 not only exhibits rapid antibacterial activity but also effectively inhibits and eradicates bacterial biofilm at a low concentration of 8 µg/mL. Additionally, when used in combination with traditional antibiotics, Cec4 enhances their antibacterial effect. Microscopy techniques, including transmission electron microscopy (TEM), confocal laser scanning microscopy, and scanning electron microscopy (SEM), found that Cec4 destroyed bacteria’s cell membrane integrity and increased the membrane permeability (flow cytometry instrument technology further characterization of Cec4 against *K. pneumoniae* bacteria antibacterial effect). Furthermore, *in vitro* experiments demonstrated that Cec4 binds to bacterial DNA and RNA of CRKP. Moreover, *in vivo* studies using a mouse skin wound model confirmed the efficacy of Cec4, and transcriptomic analysis shed light on the molecular mechanisms underlying its antibacterial activity. Based on our findings, Cec4 appears to be a promising candidate for combating CRKP infections.

**IMPORTANCE:**

The rapid increase in carbapenem-resistant *Klebsiella pneumoniae* (CRKP) infections and the serious cross-resistance to multiple antibiotics make the development of new therapeutic drugs urgent. Antimicrobial peptides (AMPs) have attracted much attention as a potential option for the next generation of antibiotics. Previous studies have identified the antimicrobial peptide Cecropin-4 (Cec4), and this study further explored its antimicrobial mechanism against CRKP. Studies have found that Cec4 shows high antibacterial activity at low concentrations, can inhibit and eradicate bacterial biofilms, and can also enhance the efficacy of traditional antibiotics. Its mechanism of action, such as destroying cell membranes and binding nucleic acid, has been revealed by various techniques, and its effectiveness has been confirmed *in vivo*, providing a promising candidate drug for combating CRKP infection.

## INTRODUCTION

The widespread use of antibiotics has led to a concerning public health issue known as antimicrobial resistance (1), and the all-cause mortality rate due to resistance-associated infections has been reported to be 6.5 deaths per 100,000 in Australia and 27.3 deaths per 100,000 in Western sub-Saharan Africa (1, 2). Among the leading causes of nosocomial infections are the multidrug-resistant ESKAPE pathogens: *Enterococcus faecium*, *Staphylococcus aureus*, *Klebsiella pneumoniae*, *Acinetobacter baumannii*, *Pseudomonas aeruginosa*, and *Enterobacter* spp. (3). Among these, carbapenem-resistant *K. pneumoniae* (CRKP) is classified as a key priority bacterium, and carbapenem-resistant *Enterobacteriaceae* is classified as a Critical Emergency by the Centers for Disease Control (4) (https://www.cdc.gov/cre/about/?CDC_AAref_Val=https://www.cdc.gov/hai/organisms/cre/).

Carbapenem antibiotics are the last treatment option for multidrug-resistant bacterial infections. However, the prevalence of *K. pneumoniae* resistant to carbapenem antibiotics is now widespread across various countries, ranging from 46% to 66% (5). Carbapenem resistance of *K. pneumoniae* strains is caused by mutations in metallo-β-lactamases, carbapenem-hydrolyzing oxacillinases, and efflux pump proteins (6). In the face of carbapenem resistance, treatment options usually involve the use of tigecycline and colistin. Unfortunately, the rate of tigecycline resistance is rising rapidly (7, 8). In instances of carbapenem resistance, colistin remains the sole course of action. Nonetheless, approximately 50% of patients receiving colistin therapy experience acute kidney injury (9, 10). Due to the scarcity of treatment options, there is an immediate need for novel antibiotics that specifically target CRKP, as well as ESKAPE pathogens in general. Antimicrobial peptides (AMPs), often referred to as host defense peptides, are produced by the host organism as a result of innate immune defenses that are ubiquitous across all life forms (11, 12). Due to their distinct mechanism of action, lack of detrimental effects on the human body, and increasing resistance from bacterial strains, AMPs are viewed as promising candidates for the development of novel, reliable, and sustainable antimicrobial therapies. Since the initial identification of the antimicrobial peptide cecropins in 1980 (13), there has been a growing academic interest in this area. To date, more than 3,000 distinct antimicrobials derived from organisms such as insects, plants, and mammals have been investigated (14, 15). AMPs exhibit common characteristics, including their low molecular weight, positive charge, and amphiphilicity in hydrophobic environments. Van et al. (16) demonstrated the antimicrobial activity of the HILL-CEC family of AMPs against *Escherichia coli* and *P. aeruginosa* at low concentrations. Additionally, Nagarajan (17) showed enhanced antibacterial efficacy against Gram-negative bacteria. These results indicate that AMPs possess promising potential as therapeutic alternatives to antibiotic treatment for bacterial infections. Given the promising potential of antimicrobial peptides in the clinical treatment of bacterial infections, Cecropin-4 (Cec4) has been shown to have potent effects on Gram-negative bacteria (18). Studies have also confirmed that modification and truncation of Cec4 resulted in MIC values ranging from 4 to 8 µg/mL for multi-resistant and extensively resistant *K. pneumoniae*. Additionally, Cec4 has been found to be non-hemolytic to human erythrocytes at high concentrations (100 × MIC) (19).

Although previous studies have demonstrated the antimicrobial efficacy of Cec4 against multi-resistant *A. baumannii*, its effect on *K. pneumoniae* is not yet known due to the significant differences in antimicrobial efficacy among different bacteria. So, the present study aimed to examine the potential of Cec4 as an antibacterial agent against clinical CRKP strains. The preliminary efficacy of Cec4 was evaluated using *in vitro* experiments, and the underlying antibacterial mechanisms were investigated through analysis of changes in bacterial cell membranes, intracellular substances, and transcriptome levels following treatment with antimicrobial peptides. Moreover, the therapeutic potential of Cec4 was examined using a mouse wound infection model. The findings from this study offer valuable insights for the development of effective anti-infective therapies for CRKP infections and may serve as a basis for the discovery of novel antibiotic drugs.

## MATERIALS AND METHODS

### Bacterial strains and growth conditions

The *K. pneumoniae* standard strain NCTC9633 (purchased from ATCC, USA, No. 13883) was maintained by the Experimental Center of the School of Biology and Engineering, Guizhou Medical University. CRKP strains were isolated from a tertiary hospital in Guizhou. All the strains were incubated overnight at 37°C in Luria-Bertani medium (LB, Sigma, USA).

### Peptide synthesis

In this study, the Cec4 antimicrobial peptide (sequence: GWLKKIGKKIERVGQNTRDATIQAIGVAQQAANVAATLKGK) was synthesized in the form of trifluoroacetic acid (TFA) salt. The peptide was synthesized by a commercial company. The company provided a report that verified and measured the relevant information of the peptide. For details, please refer to the figure. The results showed that the purity of the Cec4 antimicrobial peptide was greater than 95%, and the TFA residual amount was less than 0.5%. These data indicate that, despite the high cytotoxicity of TFA, strict purification processes allowed us to control the TFA residual amount at a low level, thereby ensuring the safety and effectiveness of the peptide in cellular and *in vivo* experiments. Cec4 was dissolved in double-distilled H_2_O (ddH_2_O) at a final concentration of 10 mg/mL. The hydrophobicity of Cec4 was analyzed using the online software HeliQuest (https://heliquest.ipmc.cnrs.fr/). The 3D spatial structure of Cec4 was predicted via the I-TASSER (https://zhanggroup.org/I-TASSER/).

### Rapid bactericidal efficiency of Cec4 against CRKP *in vitro*

#### Minimum inhibitory concentration determination

The MICs of all drugs were determined by the micro-broth dilution method according to CLSI 2018 guidelines with reference to the method of Wiegand et al. (20, 21). A bacterial suspension of 1.0 × 10^6^ colony-forming units (CFUs) per milliliter was mixed with the drug solution in a sterilized 96-well microtiter plate (NEST Biotechnology, China) and then incubated at 37°C for 16–18 h. The blank control is Mueller-Hinton Broth (MHB, Solarbio, Beijing, China) broth medium without any drugs or bacterial suspensions. The MIC value was defined as the minimum drug concentration without visible bacteria. To assess the role of ROS production in the antibacterial activity of Cec4, increasing concentrations of N-acetylcysteine (NAC) from 0 to 7.625 mM were added to the culture medium, followed by the MIC test.

#### Resistance development studies

According to the reference method (22), *K. pneumoniae* strains were selected as parents and cultured continuously for 20 generations at a concentration of 0.5 × MIC. The changes in the MIC of each generation were recorded and compared with those of their parents.

#### Time-dependent killing

According to the reference method (23), *K. pneumoniae* bacterial suspensions in the logarithmic growth phase were diluted to 1 × 10^6^ CFU/mL in LB medium. In 96-well flat-bottom plates, different concentrations of Cec4 (0–16 µg/mL) were added, mixed with the bacterial suspension, and then incubated at 37°C for 0, 0.5, 1, 1.5, 2.0, 4.0, and 6.0 h, respectively. At each time point, 10 µL of the above mixture was removed and mixed with 90 µL phosphate buffer saline (1 × PBS, 0.01 M, pH 7.2 to 7.4). Subsequently, 10-fold serially diluted suspensions were plated on LB plates and incubated overnight at 37°C. The bacterial colonies were counted, and the primary CFUs/mL was calculated.

#### Checkerboard assays

The synergistic effects of AMPs and antibiotics were measured using checkerboard assays (24). Bacterial suspensions (1 × 10^6^ CFU/mL) were added to 96-well plates (100 µL/well), and then a series of concentrations (0–8 µg/mL) of Cec4 and antibiotics (vancomycin, rifampin, levofloxacin, gentamicin, tetracycline, and ciprofloxacin) were added. MICs were recorded after 18–24 h of incubation at 37°C. The fractional inhibitory concentration index (FICI) was calculated as follows: FICI = MIC_A_ (combination)/MIC_A_ (alone) + MIC_B_ (combination)/MIC_B_ (alone), where FICI ≤ 0.5 indicates synergism, 0.5 < FICI < 1 indicates an additive effect, 1 < FICI ≤ 4 represents indifference, and FICI > 4 shows antagonism.

#### Biofilm inhibition

The ability of Cec4 to prevent biofilm formation was assessed using the crystal violet method (25, 26). In brief, *K. pneumoniae* (1 × 10^6^ CFUs/mL) with different concentrations of Cec4 (0–64 µg/mL) were cultured in a 96-well microtiter flat plate at 37°C. After a 24 h incubation, the planktonic bacteria were removed by washing three times with sterile PBS solution. Afterward, 200 µL of methanol was added, and the cells were fixed for 15 min. Next, the fixed solution was aspirated to allow for natural air drying. Dried wells were stained for 15 min with 200 µL of 0.1% crystal violet, and the remaining crystal violet was rinsed twice with PBS. Finally, 200 µL of 33% acetic acid was added and cultured at 37°C for 30 min to dissolve the crystal violet. The absorbance at 570 nm was determined as a measure of biofilm mass. The mature biofilms were constructed with 200 µL bacterial suspension (1 × 10^6^ CFU/mL) cultured for 48 h and treated with Cec4 (0–64 µg/mL) for 24 h. The biofilm was quantitatively analyzed according to the above-described method.

### Antibacterial mechanism of Cec4 against *K. pneumoniae*

#### Cec4 destroys the integrity of the cell membrane of *K. pneumoniae*

##### Molecular dynamics simulations

The molecular dynamics (MD) simulations were conducted based on previous studies with minor modifications^27^. The detailed experimental procedure is described in the Supplementary material.

##### Scanning and transmission electron microscopy

According to the reference method (28, 29), *K. pneumoniae* cells (1 × 10^8^ CFU/mL) were treated with Cec4 (16 µg/mL) for 1.5 h at 37°C before being fixed in 2.5% glutaraldehyde at 4°C overnight. The samples were dehydrated in ethanol, dried in a critical point dryer, sputter-coated with gold, and observed by scanning electron microscopy (SEM) (SU8100; Hitachi, Japan). Additionally, the samples were fixed with 1% OsO4, dehydrated in ethanol, embedded in resin, ultrathin-sectioned, stained with uranium acetate and lead citrate, and then observed using transmission electron microscopy (TEM) (HT7800; Hitachi, Japan).

##### Lipopolysaccharide and lipid inhibition assay

The effects of lipopolysaccharide (LPS, Sigma-Aldrich) and phospholipids (Sigma-Aldrich), including phosphatidylcholine (PC), phosphatidylethanolamine (PE), phosphatidylglycerol (PG), and cardiolipin (CL), on the antibacterial activity of Cec4 were evaluated using the checkerboard microdilution assay (30). Briefly, LPS (0–256 µg/mL) of *K. pneumoniae* (1 × 10^6^ CFU/mL) and phospholipids (0–521 µg/mL) were diluted twofold into the broth, and then the bacterial suspension and Cec4 mixture were added. Subsequently, the MIC test was performed to evaluate the changes in the activity of Cec4.

##### Outer membrane permeabilization

An overnight culture of *K. pneumoniae* cells was washed and resuspended in PBS (1 × 10^8^ CFU/mL), followed by the addition of 10 µM of 1-N-phenylnaphthylamine (NPN, Sigma-Aldrich) and incubation on a constant temperature shaking table at 37°C for 30 min in the dark (31). After incubation, the fluorescence intensity was detected using a Synergy H4 multifunctional microplate reader (BioTek, USA) at excitation and emission wavelengths, respectively, of 350 nm and 420 nm for 5 min. Subsequently, Cec4 (0–32 μg/mL) was added and monitored continuously for another 45 min.

##### Membrane permeability assay

The fluorescent dye propidium iodide (PI, 5 µM; Sigma, USA) was used to assess the integrity of bacterial cell membranes (32). *K. pneumoniae* cells were incubated with PI for 30 min in the dark. After incubation, the fluorescence intensity was detected using a Synergy H4 multifunctional microplate reader (BioTek, USA) at excitation and emission wavelengths, respectively, of 535 nm and 610 nm for 5 min. Subsequently, Cec4 (0–32 µg/mL) was added and monitored continuously for another 45 min.

##### Bacterial culturing, staining, and imaging

Detailed experimental procedures used for confocal and flow cytometry observations after Cec4 treatment are described in the Supplementary material.

### Bacterial membrane potential assay

Membrane permeability and dissipated membrane potential (ΔΨm) of *K. pneumoniae* induced by Cec4 (0–64 µg/mL) was tested by the fluorescent dyes (10 µM) 3,3-dipropylthiadicarbocyanine iodide (DiSC_3_(5); Sigma-Aldrich, USA) according to a previous report (19). Experiments were performed in three replicates.

### Membrane fluidity assay

*K. pneumoniae* was resuspended in PBS to 1 × 10^8^ CFU/mL, and a 100 µL suspension containing 10 µM 6-dodecanoyl-N,N-dimethyl-2-naphthylamine (Laurdan, Sigma-Aldrich, USA) was added to a black microplate containing Cec4 (0–64 µg/mL) or benzyl alcohol. After incubation for 1.5 h, the fluorescence was detected at wavelengths of 350 nm for excitation and 435 nm for emission. The Laurdan generalized polarization (GP) was calculated: GP = (I_435_ − I_490_)/(I_435_ + I_490_), where I_435_ and I_490_ show fluorescence intensities at 435 and 490 nm, respectively (1, 33).

### Reactive oxygen species measurements

Reactive oxygen species (ROS) measurements were conducted according to Shi et al. (30, 34) with some modifications. To monitor the levels of ROS in *K. pneumoniae*, 2,7-dichlorodihydrofluorescein diacetate (DCFH-DA, Yuanye Bio-Technology, China) was applied. DCFH-DA (10 µM) was pre-incubated with *K. pneumoniae* cells for 30 min, and then excess fluorescent probes that had not entered the cells were removed by centrifugation and washing with PBS. Subsequently, the probed cells were incubated with varying concentrations of Cec4 (0–64 µg/mL) for 1.5 h in the dark. The fluorescence intensity was detected at excitation and emission wavelengths of 488 and 525 nm, respectively.

### DNA gel retardation

DNA gel retardation was conducted according to a previous method with slight modifications (31, 35). *K. pneumoniae* genomic DNA was extracted using a bacterial genomic DNA extraction kit (TaKaRa, China). An equal volume of DNA was mixed with Cec4 (0–1,024 µg/mL), incubated for 1.5 h, and then electrophoresed in 1% agarose (120 V, 30 min). DNA migration was observed with a gel imaging system and photographed.

### RNA gel retardation

The assay was conducted according to a previously published method (36, 37). The total RNA of *K. pneumoniae* was prepared using an RNA prep pure Cell/Bacteria Kit according to the manufacturer’s instructions (Tiangen Biotech Co., Ltd., Beijing, China). Cec4 was dissolved in diethylpyrocarbonate (DEPC)-treated water to a final concentration of 0–1,024 µg/mL. A total of 5 µL of Cec4 solution and the same volume of bacterial RNA were mixed and incubated at room temperature for 10 min. The mixture with DEPC water instead of NP-6 was set as a control. After 1% agarose gel electrophoresis (120 V, 30 min), the migration of RNA was observed with a gel imaging system and photographed.

### Therapeutic effects of Cec4 on a CRKP infection wound model

*In vivo* experiments were described using previously published methods (38). BALB/c female mice (6–8 weeks) were purchased from SPF Biotechnology Co., Ltd. (Beijing, China). All animal studies were conducted according to the guidelines of the animal welfare system and approved by the Animal Ethics and Use Committee of Guizhou Medical University (No. 2000057).

All mice were randomly divided into two groups (*n* = 6), including a blank group (PBS-treated) and a Cec4-treated group (treated with 10 mg/kg Cec4 solution). Mice were anesthetized by inhalation of isoflurane gas, and then the hair around the surgical site was removed with an animal shaver and shaving cream. A soldering iron was applied to the skin on both sides of the mouse’s back, creating two circular wounds 1.0 cm in diameter. Then, 40 µL of CRKP100 suspension (1 × 10^8^ CFU/mL) was injected into each wound on day 0 to establish the infection model. Starting 24 h later, the wounds were treated with 40 µL of PBS or Cec4 solution (10 mg/kg, QD) for 11 consecutive days. The mice were then fed antibiotic-free food. Photographs of the wounds were taken at the indicated time points.

According to the published method (39), the mice were euthanized following 11 days of continuous treatment. The wound tissue was removed, weighed, ground, and then immersed in LB medium at a ratio of 1:100. Subsequently, it was diluted with PBS in proportion. The 10 µL sample was retrieved from the dilution in order to assess bacterial survival using agar plates. The wound skin was then fixed in a 4% paraformaldehyde solution for over 24 h and subsequently embedded in paraffin wax. Afterward, the samples were sliced into 5-µm-thick sections using a manual rotary microtome (Leica RM 2235, Nussloch, Germany). The sections were dewaxed, rehydrated, and subjected to HE staining and Masson’s trichrome staining (Solarbio, Beijing, China). Finally, the slides were sealed with neutral resin and examined using a Pannoramic Scanner (Pannoramic P250FLSAH, 3DHISTECH).

Statistical analysis: all the data were processed and statistically analyzed with Excel, Origin 8, and GraphPad Prism 8 software, and the results are expressed as the mean value, standard deviation, and significant difference (*P* value, two-tailed *t*-test, *n* ≥ 3).

### Transcriptome analysis

In brief, overnight cultures of *K. pneumoniae* were diluted 1:100, incubated in TSB until the optical density (OD) at 600 nm was 0.5, and then treated with Cec4 (8 µg/mL) or PBS for 1.5 h (17). After incubation, cells were harvested, frozen in liquid nitrogen, and stored at −80°C. RNA extraction, RNA-Seq library construction, and RNA sequencing were performed by the Novogene Corporation (Beijing, China). Three biological replicates were set for each group.

The quality and quantity of RNA were detected using an Agilent 2100 bioanalyzer and sequenced with an Illumina NovaSeq 6000 high-throughput sequencing platform. The raw data were quality-control filtered to obtain clean reads for subsequent analysis and compared with the reference genome of *K. pneumoniae* ATCC 13883 using Bowtie 2 software. After gene expression quantification was completed, the differentially expressed genes were screened according to the criteria of *P* < 0.05 and log2 (fold change) ≥4.

### Quantitative real-time PCR analysis

The expression levels of eight genes were examined by quantitative real-time PCR (qRT-PCR) to validate the RNA-Seq results. In brief, bacterial samples were prepared under the same conditions as the transcriptomes described above, total RNA was extracted using the M5 EASY spin plus kit (Mei5 Biotechnology, China), and 1 µg RNA was reverse transcribed into cDNA with a PrimeScript RT reagent kit (TaKaRa, China). According to the SYBR premix Ex Taq kit (TaKaRa, China) protocol, the reactions were performed on an ABI 7300 real-time PCR system. Gene expression levels were normalized to that of the *K. pneumoniae* RPOB gene, and relative expression levels were calculated using the 2^–ΔΔCT^ analysis method. The primers are listed in Table S4.

## RESULTS

### Characterization of Cec4 antimicrobial peptide

**Fig 1:**
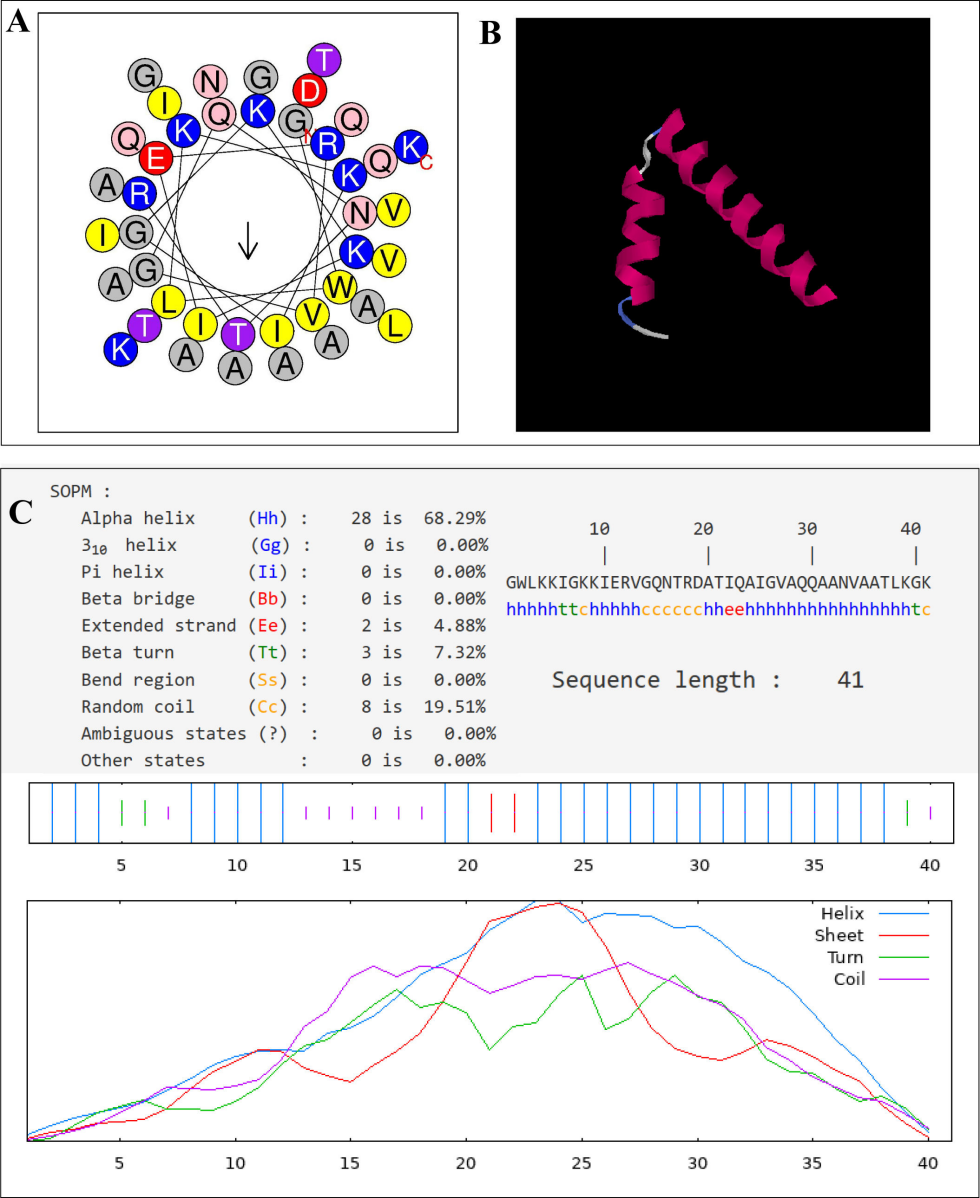
Characterization of novel antimicrobial peptide. (**A**) Helical wheel projection diagrams of Cec4 using HeliQuest analysis (https://heliquest.ipmc.cnrs.fr/). Hydrophilic amino acids are shown in blue and are positively charged. Amino acids are shown in yellow and gray are hydrophobic. (**B**) The three-dimensional structure of the peptide was predicted via I-TASSER (https://zhanggroup.org/I-TASSER/). (**C**) The secondary structure of Cec4 was predicted using the secondary structure analysis software NPS@ (https://npsa-prabi.ibcp.fr).

The results of spiral wheel analysis by the online software HeliQuest showed that Cec4 had a uniform distribution of polar and non-polar amino acids with an average hydrophobicity of 0.088 and an average hydrophobic moment of 0.594, which predicted that the peptide might form an amphiphilic structure. The secondary structure of Cec4 was analyzed by the secondary structure analysis software NPS@. The results show that Cec4 has 68.29% of α-helixes and 19.51% of random coils. The above results indicated that Cec4 exhibited typical cationic, amphiphilic, and α-helical properties (Fig. 1; Table S1).

### Cec4 effectively kills multidrug-resistant bacteria

The *in vitro* antibacterial effect of Cec4 was assessed by measuring the MIC of Cec4 against clinical isolates. The results showed that the MIC of Cec4 was 4µg/mL, 4–8 µg/mL (Table S2), and 8 µg/mL for the *K. pneumoniae* standard strain ATCC13883, 48 clinically collected CRKP strains, and two clinically collected polymyxin-resistant *K. pneumoniae* strains (Table 1), respectively.

**TABLE 1:** Strain information and MIC value of *K. pneumoniae*

Strain	Origin	MIC (μg/mL)			
		Cec4	Imipenem	Meropenem	Colistin
KP ATCC13883	–^*a*^	4	<0.125	<0.125	<0.125
CRKP100	Sputum	8	64	64	4
CRKP206	Sputum		64	128	4
CoRKP1	Sputum	4	>128	128	16
CoRKP2	Sputum	4	64	16	

^*a*^ -, the standard strain KPATCC13883 was purchased for the experiment.

**Fig 2:**
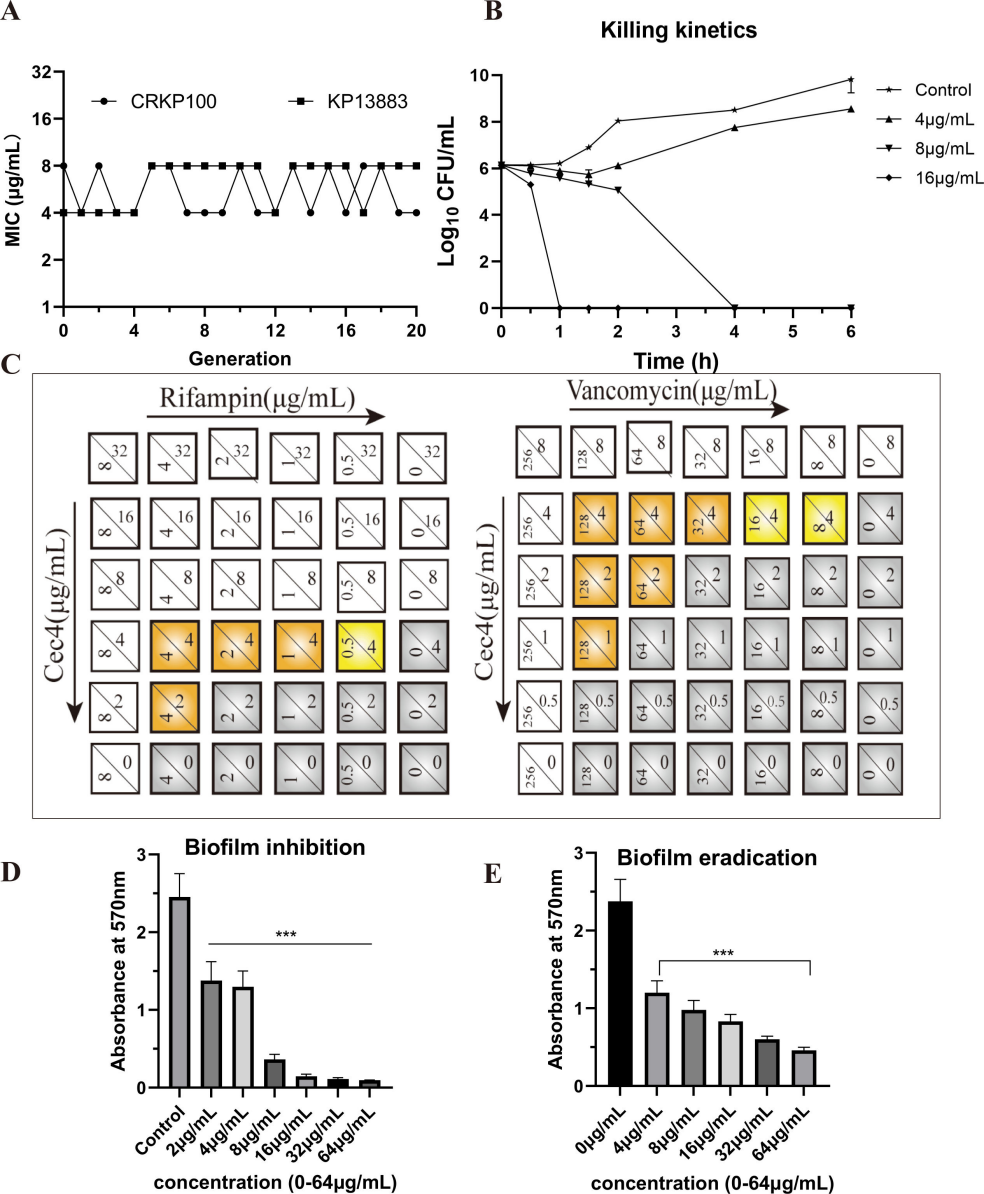
Cec4 shows bactericidal activity against *K. pneumoniae* and without detectable resistance development. (**A**) Drug resistance development of *K. pneumoniae* ATCC 13883 and CRKP 100 after sub-inhibitory dose of Cec4 treatment generations. (**B**) Time killing curve of Cec4 against CRKP 100. The experiments were conducted in triplicate and presented as means ± SD. (**C**) Checkerboard assays of Cec4 in combination with two conventional antibiotics against CRKP100. Cec4 and antibiotics were subjected to 1/2 dilution vertically and horizontally from the MIC concentration at the upper left corner. Yellow (FICI = 0.5) and orange (0.5 < FICI < 1) indicate a partial synergistic effect, and gray indicates growth of bacteria. We defined MIC as inhibiting completely over 99% of CRKP 100 bacterial growth. (**D**) Effects of Cec4 on biofilm formation (**D**) and established biofilm (**E**) of CRKP 100. Results were presented as mean ± SD. *P < 0.05, **P < 0.01, ***P < 0.001.

In the *in vitro* resistance induction assay, the MIC values of *K. pneumoniae* fluctuated 1–2-fold after 20 generations of continuous treatment with a sublethal concentration (0.5 × MIC) of Cec4, suggesting that it is not easy to induce bacterial resistance in the case of long-term use of Cec4 (Fig. 2A).

The results of timed bactericidal kinetics (Fig. 2B) showed that Cec4 had a rapid bactericidal effect in a dose-dependent manner. At concentrations of 1 × MIC and 2 × MIC, the colony number decreased by 0.43Log_10_ CFU/mL and 0.93Log_10_ CFU/mL, respectively, in 30 min. The germicidal efficacy was dose-dependent, and the bactericidal effect was achieved in 4 and 1 h, respectively.

The results of combined drug sensitivity analysis showed that when Cec4 was used with vancomycin and rifampicin, the FICI ranged from 0.5 to 1, indicating that both antimicrobial drugs had at least doubled in their activity, demonstrating an additive effect. However, when Cec4 was combined with levofloxacin, ciprofloxacin, gentamicin, or tetracycline, the FICI remained between 1 and 2, indicating no correlation between the antibacterial activity of the two drugs. These findings suggest that Cec4 may have different effects on various antibiotic combinations and warrant further investigation for the development of optimal treatment strategies for CRKP infections (Fig. 2C; Table 2).

**TABLE 2:** FICI for the synergistic effect of Cec4 in combination with antibiotics against CRKP

Cec4 with antibiotics	MIC (μg/mL)		MIC (μg/mL) in combination		FICI
	Cec4	Antibiotics	Cec4	Antibiotics	
Cec4+rifampin	8	8	4	0.5	0.56
Cec4+vancomycin	8	256	4	8	0.50
Levofloxacin	8	128	8	128	2
Gentamicin	8	>256	8	>256	2
Tetracycline	8	256	8	256	2
Ciprofloxacin	8	128	8	128	2

The results for inhibition and clearance of biofilm showed that biofilm formation in *K. pneumoniae* contributes to its tolerance and resistance to antibiotics. The results for the inhibitory and dispersing effects of Cec4 on biofilm formation showed that Cec4 at concentrations of 2 µg/mL (0.5 × MIC) or higher can significantly decrease biofilm formation ability (*P* < 0.001). In addition, the mature biofilm was treated with Cec4, and the amount of biofilm decreased significantly after the mature biofilm was treated with 1–16 × MIC Cec4. In addition, when mature biofilms are treated with 1–16 × MIC of Cec4, their biofilm mass decreases significantly (Fig. 2D and E). These findings suggest that Cec4 may have potential as a therapeutic agent for treating bacterial infections caused by biofilm-forming bacteria.

**Fig 3:**
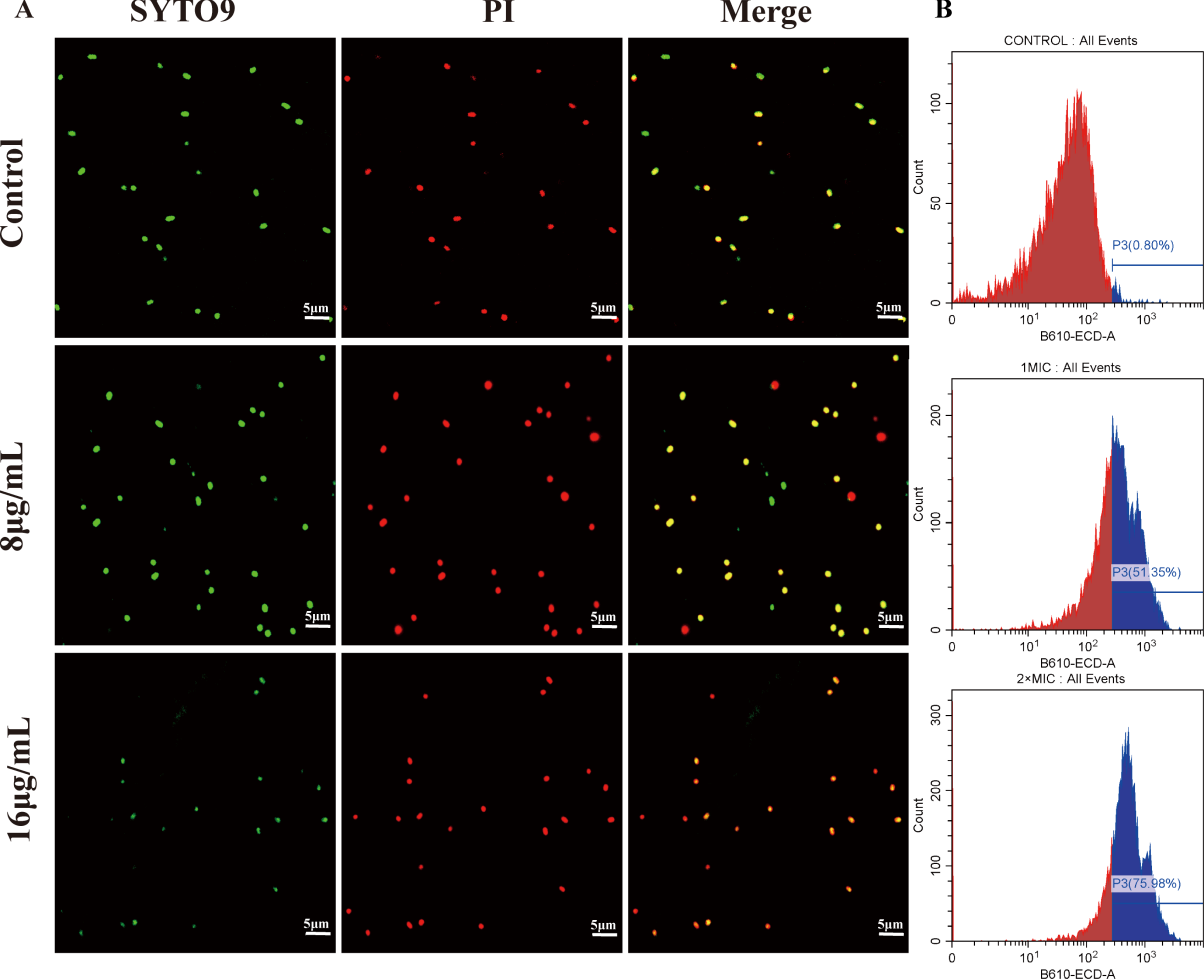
Killing capacity of Cec4 with CRKP100. (**A**) Bacterial survival after 1.5 h of treatment with different concentrations of Cec4 (8–16 μg/mL) was observed using laser scanning confocal microscopy. (**B**) Bacterial survival after 1.5 h of treatment with different concentrations of Cec4 (8–16 μg/mL) was observed using flow cytometry. Without the addition of antimicrobial peptides, the samples represent a negative control. Red fluorescence indicates dead bacteria, green and yellow fluorescence indicate viable bacteria.

To further determine the change in membrane damage caused by Cec4, the death of *K. pneumoniae* after Cec4 treatment was evaluated by confocal laser scanning microscopy with an index that could penetrate the damaged cell membrane and insert PI dye and the green fluorescent nucleic acid stain SYTO 9 Green Fluorescent Nucleic Acid Stain (SYTO9, Sigma, USA). Because the fluorescent nucleic acid stain SYTO 9 and fluorescent nucleic acid dye PI have different spectral characteristics and differ in their ability to penetrate bacterial cells, SYTO 9 usually marks all bacteria in the population, while PI only penetrates damaged bacteria. The results showed that when the concentration of Cec4 increased from 4 to 8 μg/mL, bacterial mortality increased (Fig. 3A). Similarly, according to the principle of PI penetration, bacterial death was enumerated by flow cytometry (Fig. 3B). When the concentration of Cec4 increased from 1 to 2 × MIC, the mortality in the population increased from 59.06% to 75.48%.

### Cec4 triggers membrane damage in CRKP bacteria

**Fig 4:**
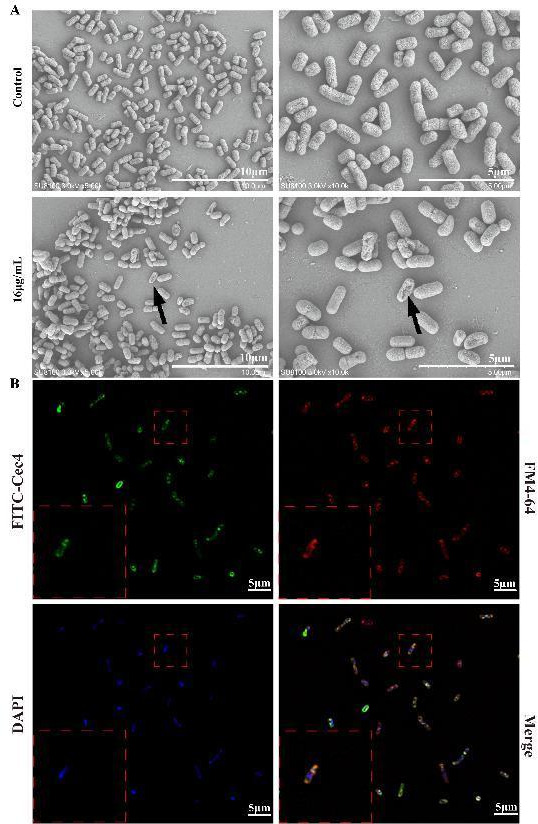
Membrane active mechanism of action by Cec4. (**A**) SEM images of CRKP100 treated with Cec4 (16 µg/mL) for 1.5 h. Black arrows exhibited wrinkling, collapsed, and lysed structures on the cell membrane. (**B**) Confocal laser scanning microscopy (CLSM) shows fluorescein isothiocyanate (FITC)-labeled Cec4-treated *K. pneumoniae* ATCC 13883. Cec4 colocalized with FM4-64, indicating a membrane-binding propensity at 1 × MIC for 1 h. The scale bar in this figure is 5 µm.

For the powerful antibacterial activity of Cec4, this study investigated its membrane mechanism from several aspects. To begin with, we observed morphological and structural changes in bacteria after peptide Cec4 treatment. The results indicated that untreated *K. pneumoniae* appeared as short rods with a smooth and dense surface (Fig. 4A). However, after treatment with Cec4 at 16 µg/mL (2 × MIC) for 1.5 h, the structure loosened, and the integrity of the membrane was disrupted. Changes such as perforation, wrinkling, collapsing, and dissolution appeared on the surface of the membrane. At the same time, using *K. pneumoniae* ATCC 13883 as a model, the bacterial cell membrane and nucleus were cooperatively labeled with the commercial dye N-(3-triethylammoniumpropyl)-4-(6-(4-(diethylamino) phenyl) hexatrienyl) pyridinium dibromide (FM4-64, Sigma, USA) and DAPI (4′,6-diamidino-2-phenylindole, Sigma-Aldrich) (Fig. 4B). The result of TEM can strongly indicate the membrane-active mechanism of Cec4. We found that the whole cell was stained, especially on the cell membrane, where the fluorescence intensity was the strongest. This indicates that Cec4 has been effectively accumulated in *K. pneumoniae*.

**Fig 5:**
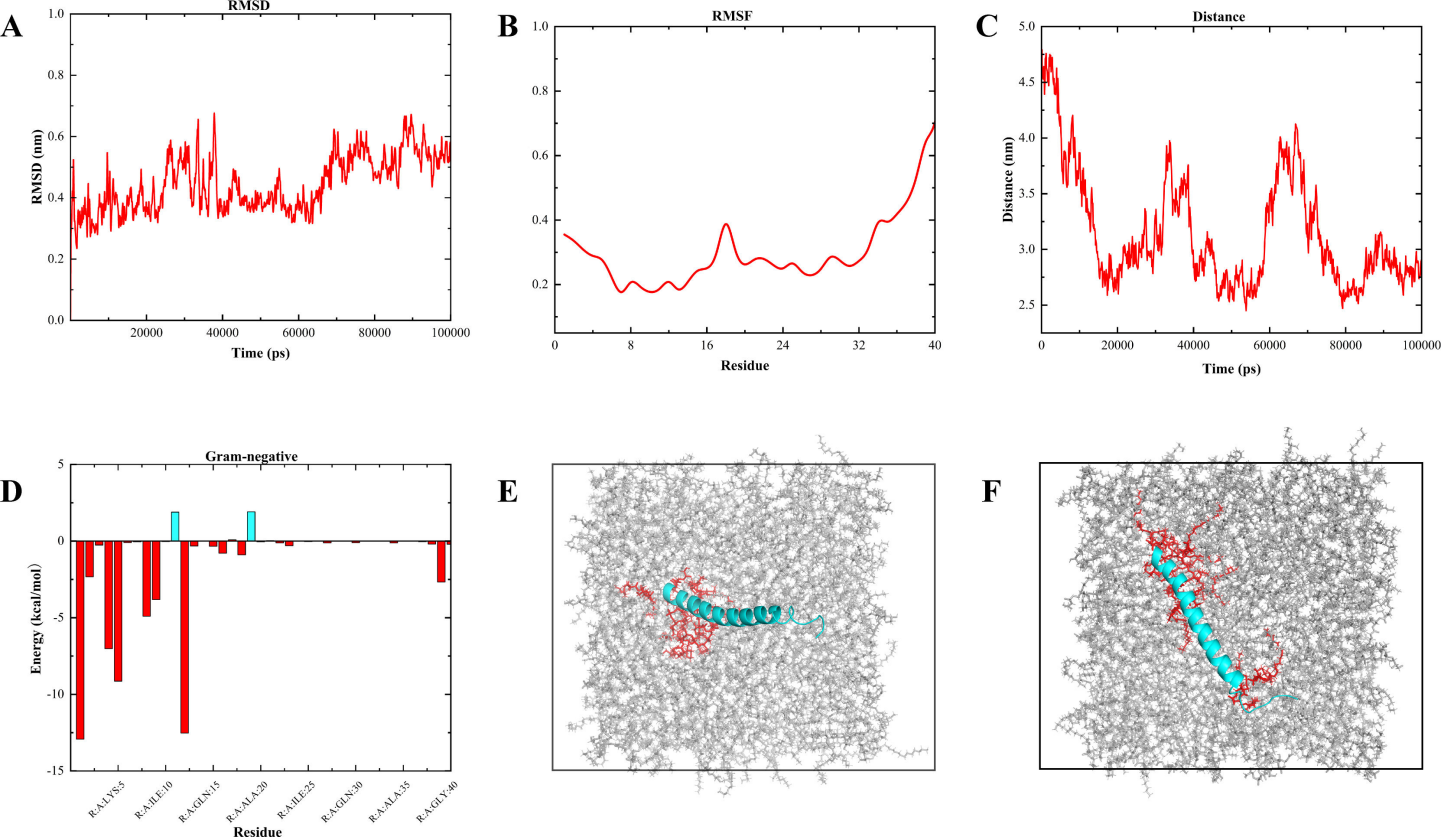
Interaction of Cec4 and Gram-negative bacterial membranes in a molecular dynamics simulation system. (**A**) The root-mean-square deviation (RMSD) value of Cec4. (**B**) The root-mean-square fluctuation (RMSF) value of Cec4. (**C**) Distance from each amino acid to the centre of mass of the bacterial membrane. (**D**) Binding energy contributions of Cec4 residues to Gram-negative bacteria. (E and F) MD simulation images of Cec4 interacting with a 3POPG: 1POPE lipid bilayer at representative time points (10 and 100 ns).

Next, we further simulated the interaction of Cec4 with the phospholipid bilayer of the cell membrane of Gram-negative bacteria using MD. During the 100 ns simulation, the MD simulation system constructed in this study was in a steady state (Fig. S1), and the average values of root-mean-square deviation (RMSD), root-mean-square fluctuation (RMSF), and distance of Cec4 in the phospholipid bilayer were 0.445, 0.315, and 3.150 nm, respectively (Fig. 5A through C). The binding free energies of each amino acid are shown in Fig. 5D, and the total binding free energy was −55.36 kJ/mol. Meanwhile, we observed at 10 ns that residues of protein Gly1, Lys5, Lys8, Lys9, Arg12 can and part of the membrane lipid (red) interact. At 100 ns, the vertical orientation of the protein gradually changed to the horizontal orientation, and the residues on both sides of the N and C termini could interact with the bilayer membrane: Gly1, Lys5, Lys8, Arg12, Lys39. The main type of interaction is electrostatic interaction between positively charged side chains of protein residues and negatively charged lipid membranes. (Fig. 5E and F).

**Fig 6:**
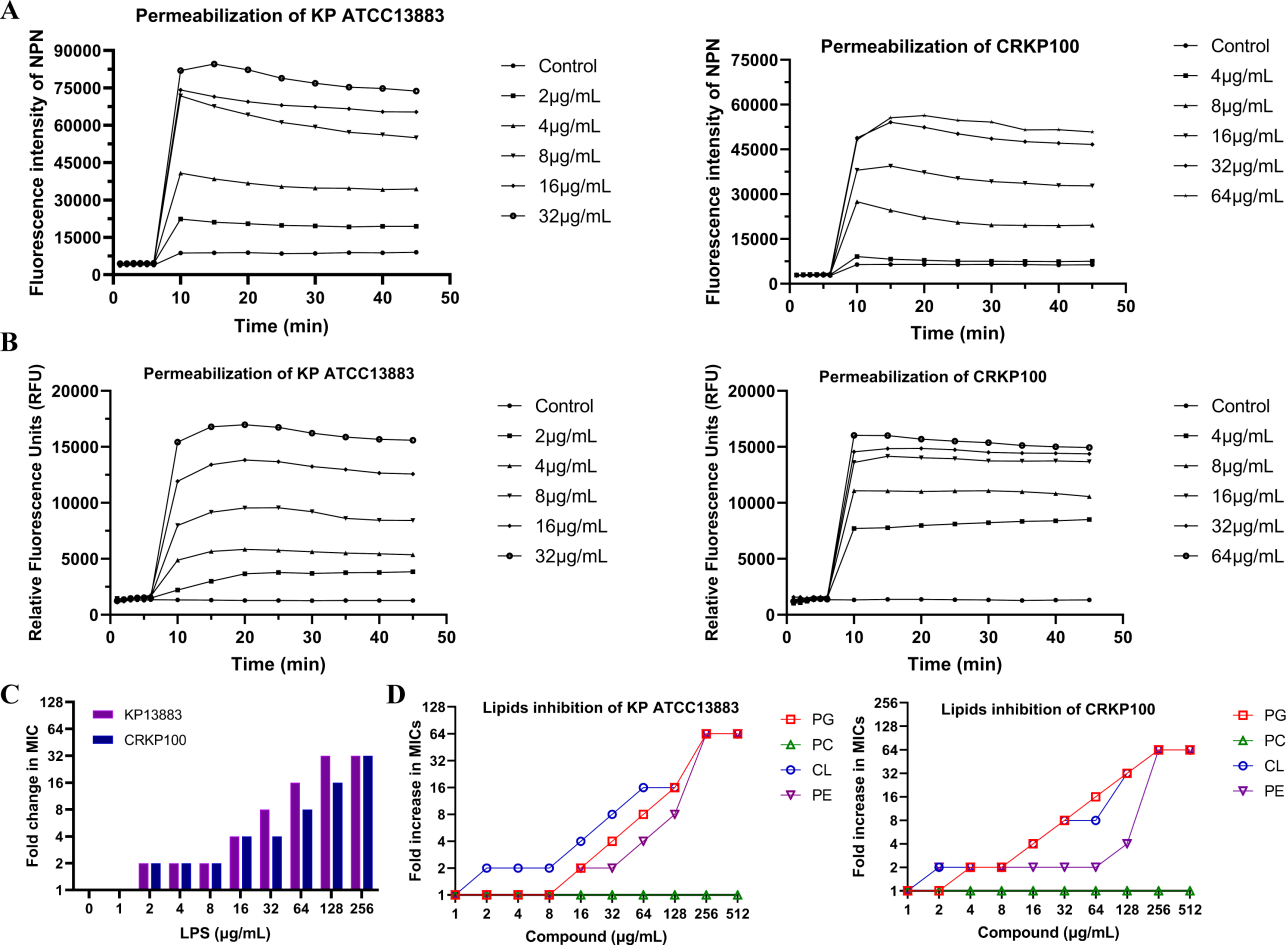
Cec4 binds to cell membrane components and alters membrane permeability. Whole cell membrane permeability of *K. pneumoniae* ATCC 13883 and CRKP 100, which were assessed by fluorescence probes NPN (**A**) (excitation 350 nm and emission 420 nm) and PI (**B**) (excitation 535 nm and emission 615 nm), respectively. CLSM shows Cec4 in *K. pneumoniae* ATCC 13883 and CRKP 100 after treatment with Cec4 at 8–16 μg/mL for 1.5 h. (**C**) Exogenous addition of LPS impaired the antibacterial activity of Cec4 against *K. pneumoniae* ATCC 13883 and CRKP 100 in a dose‐dependent manner. (**D**) Increased MICs of Cec4 against *K. pneumoniae* ATCC 13883 and CRKP 100 in the presence of CL, PG, PC, and PE, ranging from 1 to 512 µg/mL.

Then, to further characterize membrane damage in *K. pneumoniae* induced by Cec4, the effect of Cec4 on outer membrane permeability in *K. pneumoniae* was evaluated with the hydrophobic fluorescence probe NPN. The results showed that the fluorescence intensity increased in a concentration-dependent manner, indicating that Cec4 seriously damaged the adventitia (Fig. 6A) of *K. pneumoniae*. Then, the permeability of the whole membrane was evaluated by PI staining. As shown in Fig. 6B, Cec4 increased the fluorescence of PI in *K. pneumoniae* in a dose-dependent manner, which means that the cell membrane of *K. pneumoniae* was significantly damaged.

Finally, we further analyzed its affinity for phospholipid components of bacterial membranes. The results suggest that the MIC value of Cec4 will be affected when exogenous LPS is added artificially, and the change in the MIC value is dose-dependent (Fig. 6C). In addition to the outer membrane, the cytoplasmic membrane is an important distinguishing feature of Gram-positive and Gram-negative bacteria. PG, CL, and PE are the main components of the cytoplasmic membrane. In order to further understand the potential targets of Cec4, we compared the antibacterial activity of Cec4 in the presence of the three main bacterial membrane phospholipids, PE, PG, and CL. The results suggest that PG, CL, and PE specifically eliminate the antibacterial activity (Fig. 6D) of Cec4 in a dose-dependent manner. These results indicate that Cec4 can specifically bind relevant components of the bacterial membrane, such as LPS, PG, CL, and PE. However, more studies are needed to clarify the further mechanism of action. In conclusion, Cec4 is a membrane-breaking antimicrobial peptide that may target relevant components of bacterial membranes in a time- and dose-dependent manner.

### Cec4 dissipates the ∆Ψ component of the bacterial proton motive force

**Fig 7:**
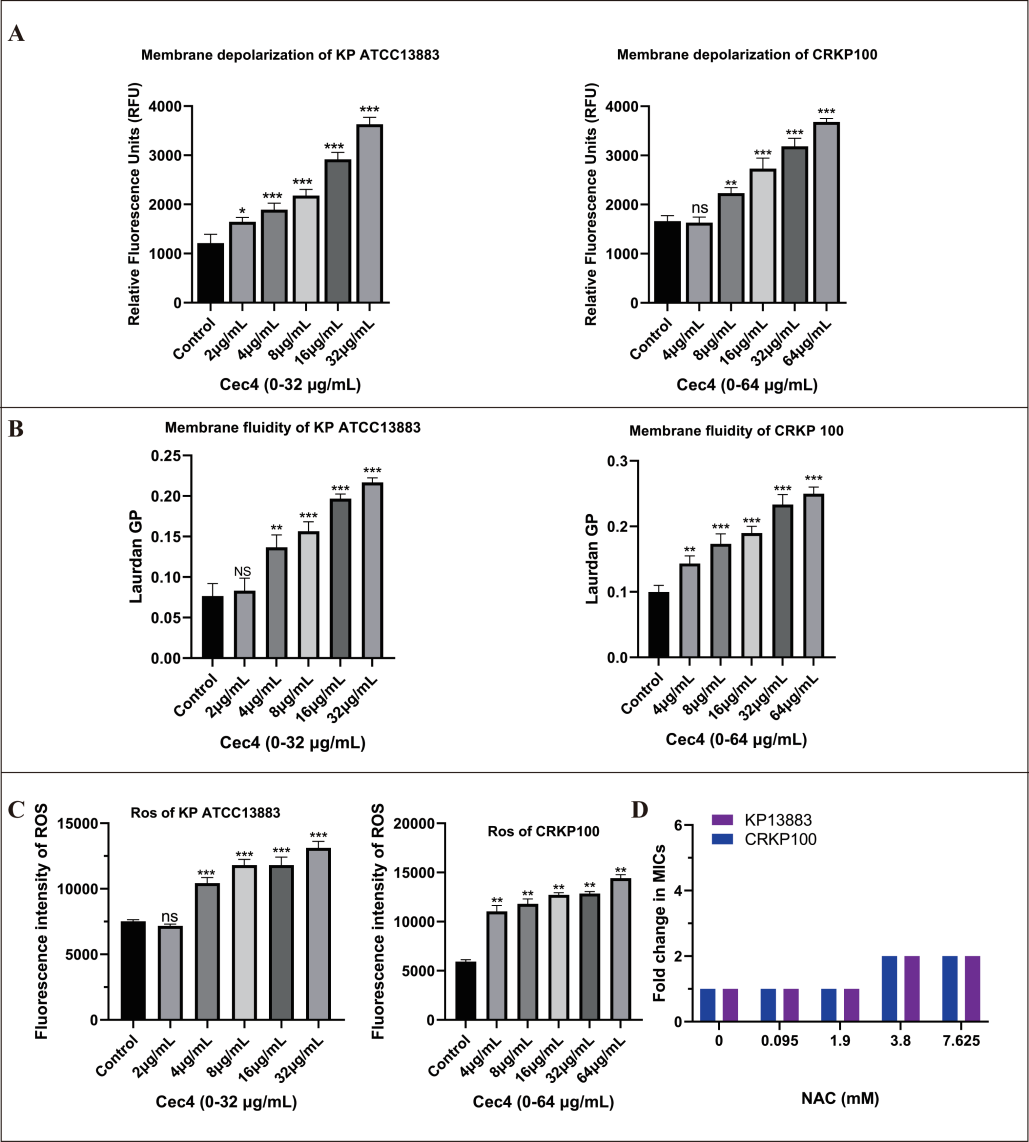
Effects of Cec4 on bacterial cell membrane and oxidative stress. (**A**) Cec4 dissipates the ΔΨ component of the PMF. Cec4 dissipated the membrane potential of *K. pneumoniae* ATCC 13883 and CRKP 100 determined by monitoring the fluorescence intensity of 3,3'-dipropylthiadicarbocyanine iodide (DiSC3(5), excitation at 622 nm and emission at 622 nm). (**B**) Membrane fluidity changes in *K. pneumoniae* ATCC 13883 and CRKP 100 under exposure to Cec4. Membrane fluidity was determined using 10 µM Laurdan, and the fluorescence intensities were detected with emission wavelengths of 435 and 490 nm upon excitation at 350 nm. (**C**) Cec4 triggers the production of ROS in *K. pneumoniae* ATCC 13883 and CRKP 100. Before the fluorescence assay, probe-labeled cells were incubated with Cec4 at 37°C for 1.5 h. (**D**) NAC supplementation abolished the antibacterial activity of Cec4 against *K. pneumoniae* ATCC 13883 and CRKP 100. All data were presented as mean ± SD, and the statistical significance was determined by non‐parametric one‐way analysis of variance (NS, no significant; results were presented as mean ± SD. *P < 0.05, **P < 0.01, ***P < 0.001).

The proton motive force (PMF) is generated by bacterial transmembrane potential and consists of two parameters: potential (∆Ψ) and transmembrane proton (∆pH). These two components work together to maintain the dynamic balance of PMF, which is essential for the survival of bacteria. To study the effect of Cec4 on the membrane potential of *K. pneumoniae*, the cell membrane potential-sensitive fluorescent dye DiSC3(5) (Sigma-Aldrich, USA) was used. DiSC_3_(5) accumulated in the plasma membrane in response to the ∆Ψ component of PMF. With the increase in the Cec4 concentration, the degraded probe will be released into the extracellular environment, resulting in fluorescence enhancement (Fig. 7A). The detection of membrane fluidity by the fluorescence polarization probe Laurdan is based on a fluorescence emission shift, which depends on the number of water molecules around the probe, which can reflect the diffusion of the lipid head group and the fluidity of the fatty acid chain and is quantified by Laurdan GP. The results showed that Cec4 induced a significant increase in Laudan GP in a concentration-dependent manner, indicating that the membrane fluidity decreased in the presence of Cec4 (Fig. 7B).

### ROS plays an important role in the bacteriostatic process of Cec4

The production of ROS has been considered a key factor in antibiotic-mediated killing. In order to further verify whether the bacteriostatic and bactericidal effects of Cec4 are enhanced by the production of ROS, we then used DCFH-DA to detect the level of ROS in bacteria after Cec4 treatment. Cec4 triggered the production of ROS in a concentration-dependent manner (Fig. 7C). In order to evaluate the effect of antibacterial Cec4 activity on the production of ROS, the active oxygen scavenger NAC was added in the following MIC analysis. Interestingly, the addition of NAC (5 mM) significantly destroyed the activity of Cec4 against *K. pneumoniae*, and the MIC increased twofold (Fig. 7D). These results suggest that the production of reactive oxygen species is the key to the killing effect of Cec4 on *K. pneumoniae*.

### Binding ability of Cec4 to bacterial DNA and RNA

**Fig 8:**
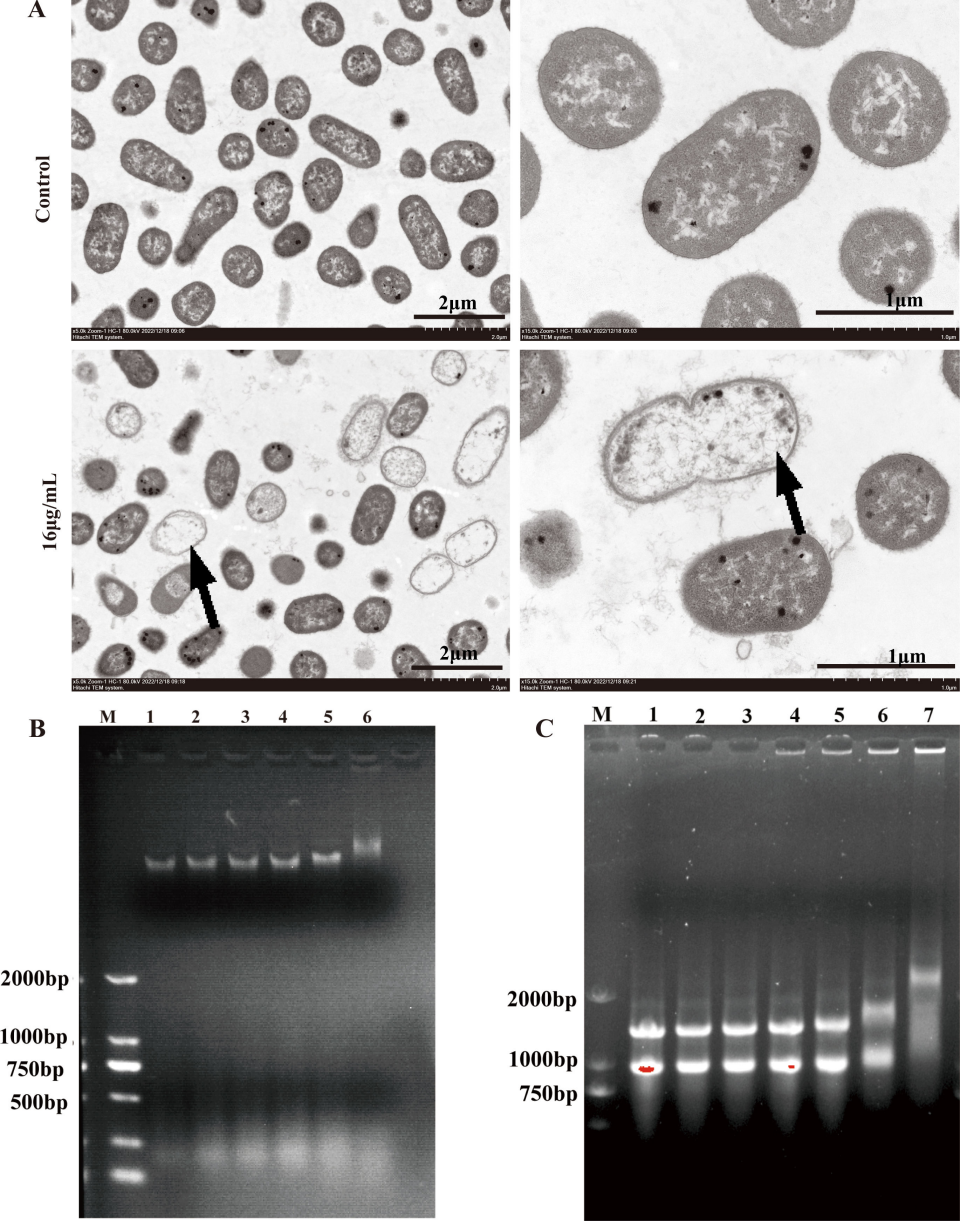
Effects of Cec4 on bacterial cell membrane and oxidative stress. (**A**) TEM images of CRKP 100 treated with Cec4 (16 µg/mL) for 1.5 h. (**B**) Gel retardation analysis of the binding of Cec4 to CRKP 100 DNA. (**C**) Gel retardation analysis of the binding of Cec4 to CRKP 100 RNA. Lane M: DL2000 DNA Marker; Lanes 1–6: the genomic DNA/RNA treated with Cec4 of 0–512 μg/mL. Lane 7: the genomic RNA treated with Cec4 at 1,024 µg/mL.

In addition to disrupting the cell membrane, binding to intracellular substances is also a key reason for the antibacterial activity of Cec4. Firstly, the changes in bacterial morphology and structure after Cec4 treatment were observed by transmission electron microscopy. The results showed that the intracellular material of untreated *K. pneumoniae* was uniform and dense, and the cell wall was visible (Fig. 8A). After treatment with Cec4 (16 µg/mL) for 1.5 h, the bacterial morphology was irregular, and the cell membrane and cell wall structures were not clear. The phenomenon of dissolution and rupture occurred, and the leakage of cell contents formed the shape of a bubble (black arrow). The binding ability of Cec4 to bacterial genomic DNA was compared *in vitro*, and a DNA gel block was performed to confirm the binding activity of Cec4 to bacterial DNA. The results showed that the brightness of DNA bands in agarose gel electrophoresis decreased as the Cec4 concentration increased (Fig. 8B). It is suggested that Cec4 has DNA-binding activity *in vitro*. The interaction between Cec4 and bacterial RNA was further evaluated using a gel block test. From the results of (Fig. 8C), it can be seen that the color of RNA bands gradually darkens as the Cec4 concentration increases. In addition, the DNA bands treated with 256 and 1,024 µg/mL Cec4 were almost trapped in the pores, indicating that Cec4 has RNA-binding activity *in vitro*.

### Therapeutic effects of Cec4 on a CRKP infection wound model

**Fig 9:**
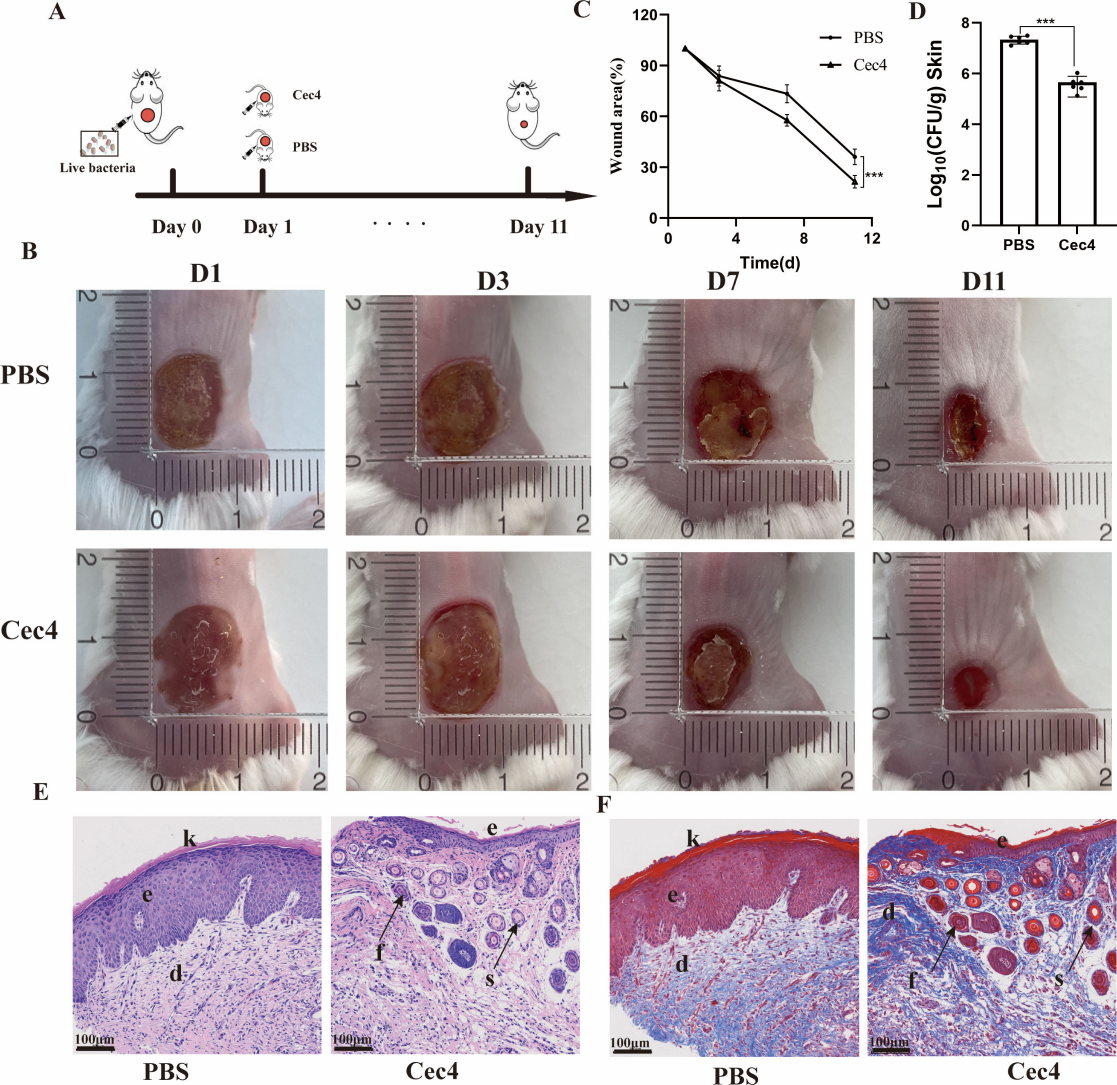
*In vivo* antibacterial assay. (**A**) Schematic illustration of the *in vivo* bacterial infection and treatment using Cec4. (**B**) Bacterial infection was treated 1 day after infection, with photographs taken on days 1, 3, 7, and 11 after treatment. (**C**) Quantification of the process of wound healing for all groups (*n* = 6). (**D**) The subeschar bacterial colonies on the 11th day. (**E**) HE and Masson-stained (**F**) histologic sections of wounds on the 11th day. Abbreviations: f, follicle; s, sebaceous gland; k, keratinized layer; e, epidermis layer; d, dermis. Original magnification: ×100. *** denotes *P* < 0.001.

CRKP is a common multidrug-resistant bacterium that is resistant to almost all antibiotics, including β-lactams, macrolides, and aminoglycosides. We used a CRKP-infected wound model in mice to study the *in vivo* therapeutic effect of Cec4 (Fig. 9A). After infection, treatment, and cultivation, wound images showed that compared to the control group (treated with PBS, Fig. 9B through D), Cec4 improved wound healing on day 7. The wound area in the PBS group was reduced to 73.34% on day 7, while in the Cec4-treated group, it was reduced to 57.70%. By day 11, the wound area in the PBS group further decreased to 36.10%, whereas in the Cec4-treated group, it decreased to 21.39% (*P* < 0.001). Moreover, the scabs fell off, and the surrounding skin of the wound became smooth and intact. Wound counting results showed a significant reduction in the bacterial count in the crusts beneath the scabs of mice in the Cec4 treatment group compared to those in the PBS group (*P* < 0.001).

Through histological analysis using HE and Masson staining, we conducted a comprehensive evaluation of the burn healing process (Fig. 9E). The figure illustrates that on the 11th day, the epidermal layer in the PBS group appeared thicker, possibly due to the presence of an unshed scab. Additionally, no skin appendages were observed. Conversely, in the Cec4 treatment group, clear observations of hair follicles, sebaceous glands, and the dermal layer were made. Masson staining, depicted in Fig. 9F, revealed the blue staining of collagen protein. Notably, after Cec4 treatment, a more dense and organized structure of collagen protein was observed within the wound. These findings strongly indicate that Cec4 plays a crucial role in promoting skin regeneration and accelerating wound healing in bacterial infection models.

### Molecular response of *K. pneumoniae* to a Cec4 challenge

**Fig 10:**
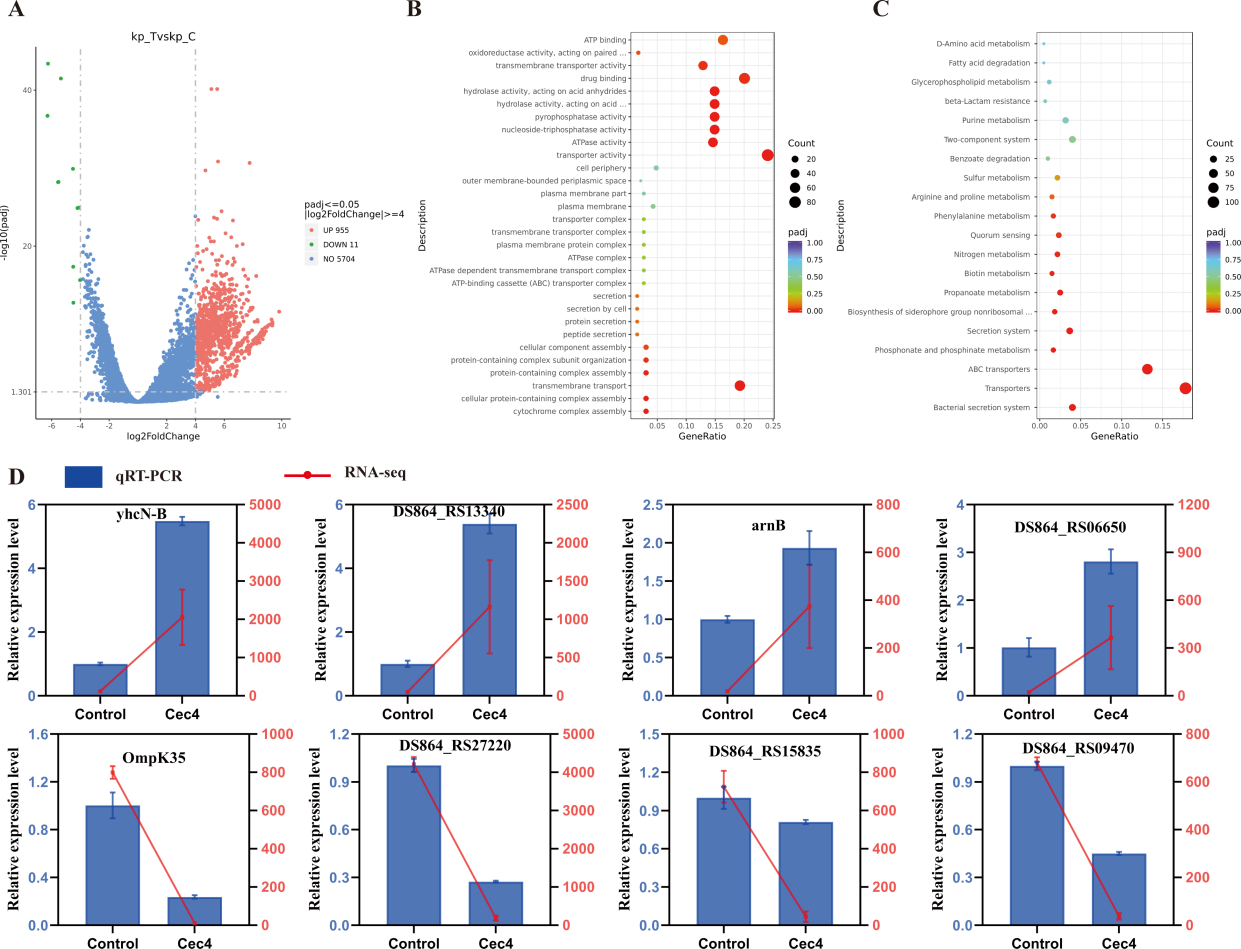
Transcriptome analysis of *K. pneumoniae* ATCC 13883 after exposure to Cec4. (**A**) Volcano plot annotation analysis of the differential expression genes (DEGs) in *K. pneumoniae* ATCC 13883 after exposing Cec4 (8 µg/mL) for 1.5 h. Significantly differentially expressed genes were treated with red dots (up-regulated) or green dots (down-regulated). The abscissa represents fold change, and the ordinate represents statistical significance. (**B**) Gene Ontology (GO) annotation analysis of the DEGs in *K. pneumoniae* ATCC 13883. (**C**) The classification of genes into Kyoto Encyclopedia of Genes and Genomes (KEGG) terms after enrichment analysis. Up-regulated genes are labeled red, and down-regulated genes are labeled green. (**D**) Comparison of eight gene expression levels between RNA-Seq and qRT-PCR. The red line represents the FPKM value of the gene in RNA-Seq, and the blue columns represent the relative expression value calculated by qRT-PCR. The experiments were performed in three biological replicates, and data are presented as means ± SD.

In order to understand the molecular mechanism of Cec4 action at 8 µg/mL and the changes in gene expression induced by mRNA, we performed transcriptional analysis of *K. pneumoniae* ATCC13883 exposed to Cec4 (8 µg/mL) after 1.5 h. The results showed that 966 differentially expressed genes were obtained, of which 955 were up-regulated and 11 were down-regulated (Fig. 10A).

The results of Gene Ontology (GO) enrichment showed that the differentially expressed genes were mainly related to biological processes, cell components, and molecular function (Fig. 10B; Table S3). Specifically, nine differentially expressed genes were up-regulated (*DS864-RS13340*, *DS864-RS26095*, etc.) and one was down-regulated (*ompK35*) among adventitia-related genes, and the intimal components were enriched to 27 (*gspF*, *phnE*, *DS864-RS00195*, *DS864-RS21610*, etc.), all of which were up-regulated, suggesting that different membrane proteins may need to be up-regulated to compensate for membrane damage induced by Cec4. A total of 13 genes related to electron transmission were significantly upregulated (*ybtU*, *DS864-RS17105*, *DS864-RS06340*, etc.).

In addition to membrane-related genes, amino acid transport, oxidative stress, and DNA and RNA-related genes in bacteria also changed after Cec4 treatment (Table S3), which was specifically reflected in the upregulation of nine amino acid transport genes (*DS864-RS06000*, *DS864-RS06005*, *gguB*, etc). Oxidative stress genes were enriched to four (*yhcN*, *yhcN-B*, *uspF*, *pspG*), which was consistent with our experimental results, suggesting that Cec4 treatment may play a bactericidal effect by inducing bacteria to produce reactive oxygen species. In addition, the genes involved in the formation of DNA (8) and RNA (8) were also significantly up-regulated, suggesting that Cec4 may inhibit bacterial growth by affecting the synthesis of intracellular DNA and RNA, which was further verified by experiments.

In the Kyoto Encyclopedia of Genes and Genomes (KEGG) database, 11 pathways were significantly enriched, including the bacterial secretory system, transport system, ABC transport system, phosphate, hypophosphite metabolism, iron carrier group of secretory system, biosynthesis of non-ribosomal peptides, and phenylalanine metabolism. The differentially expressed genes involved in these pathways were all up-regulated genes. Genes such as *DS864-RS26865* and *DS864-RS21760* were significantly up-regulated in the ABC transport system (Fig. 10C). Cell membrane (*DS864-RS13340*, *OmpK35*), oxidative stress (*yhcN-B*), nucleic acid (*arnB, DS864-RS15835*), ion transport (*DS864-RS06650*), and membrane transport protein components (*DS864-RS27220, DS864-RS09470*) were randomly selected and analyzed by qRT-PCR. The results showed that the expression of *DS864-RS13340*, *yhcN-B*, *arnB,* and *DS864-RS06650* genes was higher than that of the control group, and the expression of *OmpK35*, *DS864-RS15835*, *DS864-RS27220,* and *DS864-RS09470* genes was lower than that of the control group. In other words, the results of the transcriptome are consistent with the changes in the RNA-Seq data, which further validates our experimental results (Fig. 10D).

## DISCUSSION

The prevalent use and misuse of antibiotics has resulted in an escalation in drug resistance among numerous significant human pathogens (40). Due to its elevated infection rate and substantial mortality, CRKP has garnered attention (41). Due to its resistance to carbapenem antibiotics, the available treatment options for CRKP are limited to tetracycline and colistin. Unfortunately, both drugs have notable drawbacks. Tetracycline is facing an increasing prevalence of resistance, and colistin exhibits nephrotoxicity with long-term use (10). Cec4 is membrane active like other amphiphilic AMPs, and the antimicrobial mechanism is not much different from other AMPs (42), which is common to cecropins. But its mode of action and antimicrobial effect are different for different bacteria. For example, in the study by Shi et al. (43), the MIC value of the antimicrobial peptide LI14 was 32 µg/mL against *K. pneumoniae*, but only 8 µg/mL against *A. baumannii*, which shows that the antimicrobial mechanism of the antimicrobial peptide has not been well-studied. Therefore, although Cec4 exhibited antibacterial activity against multidrug-resistant *A. baumannii* (MIC = 8 µg/mL), its antibacterial effect against CRKP was not found. Based on the previous findings, Cec4 and its derived peptides are not only resistant to the humoral environment, including ions, serum, and proteases, but they also exhibit low toxicity and high safety *in vivo* (44).

In this study, Cec4 was identified as a potential α-helical peptide with significant inhibitory activity against CRKP strains based on structural prediction, with a MIC of 8 µg/mL. Furthermore, Cec4 exerted a rapid bactericidal effect within 30 min, and its bactericidal rate was dependent on the dose. There was no significant difference in the number of killed *K. pneumoniae* colonies after the addition of Cec4 solution at concentrations of 4 and 8 µg/mL in the first 2 h. This is the same as in the study by Alys et al (45). and may be due to the “inoculum effect” (dependence of MIC on initial inoculum concentration). *In vitro* induction experiments revealed no significant development of drug resistance after 20 generations of continuous induction with subinhibitory concentrations of antimicrobial peptides. Indeed, the observation of resistance with subinhibitory concentrations induced for 20 generations takes into account only brief episodes of selection and cannot exclude the evolution of resistance through cumulative changes involving multiple loci (46). Therefore, more in-depth studies should be conducted to observe the evolution of resistance in Cec4. In clinical settings, diffractive biotics are often combined to broaden the antibacterial spectrum. The combination of Cec4 with rifampicin and vancomycin has been found to enhance the bacteriostatic effect, consistent with the findings of Choi et al. (47). However, the specific mechanism behind this synergy remains unclear. Biofilms are membrane structures formed by bacteria when growing in hostile environments. They adhere to surfaces or gas-liquid interfaces and consist of microcolonies as their fundamental unit. Biofilm formation enables bacteria to evade the host immune system and persist within the host, providing an optimal growth environment (48). Consequently, we investigated the impact of Cec4 on the formation and elimination of *K. pneumoniae* biofilms. Our results showed that Cec4 possessed inhibitory and scavenging abilities against *K. pneumoniae* biofilms. Furthermore, confocal microscopy and flow cytometry confirmed the antibacterial activities of Cec4 on *K. pneumoniae.*

**Fig 11:**
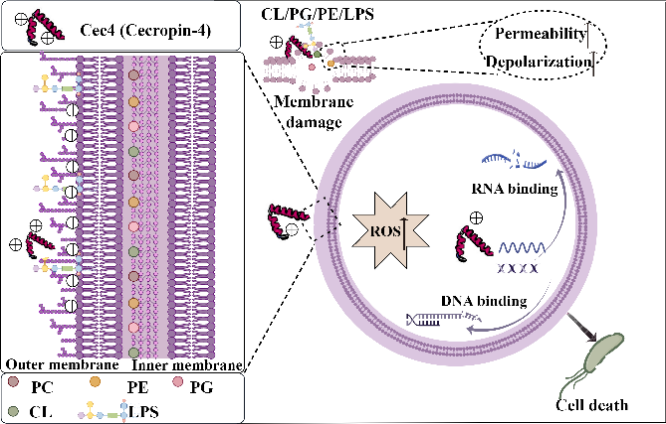
Schematic representation of the mode of action of Cec4 against *K. pneumoniae.* Cec4 may kill the *K. pneumoniae* by crossing the cell wall and destroying cell membrane integrity, further resulting in membrane dysfunction, or acting on intracellular targets, to affect the transcriptional regulation of DNA and ribosomal biosynthesis, which in turn affects the normal physiological activities of bacteria. The picture was drawn with Figdraw.

With regard to the mechanism of action of Cec4, it has been found that Cec4 functions as a peptide antibiotic that acts on the cell membrane. Most AMPs, particularly cationic AMPs, exert bactericidal effects by disrupting the integrity of the cell membrane. On this basis, there are three commonly used modes of action to describe the membrane activity of AMPs: the bucket plate, carpet, and ring models (49). The cell membrane, which acts as an important barrier for Gram-negative bacteria in resisting the external environment, plays a crucial role in bacterial respiration and energy metabolism (50). The integrity of the cell membrane is the key factor in its ability to function as a barrier. In this study, the damage caused by Cec4 on the cell structure was demonstrated visually through the use of scanning electron microscopy and transmission electron microscopy. These techniques revealed changes in cell morphology, dissolution of the cell membrane, perforations, and an unclear cell wall structure. Additionally, confocal observation following the labeling of Cec4 with FITC suggested that Cec4 may exert its bacteriostatic and bactericidal effects by interacting with the cell membrane of *K. pneumoniae*. To verify this hypothesis, the interaction between Cec4 and the main phospholipid components of the membrane, namely PG, PC, CL, and PE (Fig. 11), was studied. Indeed, we found differences in the effect of the action of Cec4 and different membrane lipid components, which may be due to the fact that cholesterol affects the affinity of the antimicrobial peptide for the membrane, as shown by Yu et al (51). The results indicate that the phospholipids within the membrane may serve as potential sites of action for Cec4. Subsequently, the permeability of the cell membrane, level of depolarization, membrane fluidity, and increase in reactive oxygen species were assessed after treatment with Cec4 in order to further investigate its effects. In our current understanding, Cec4’s antimicrobial activity plays a significant role in its ability to reduce biofilms. Regarding the specific mechanism of biofilm reduction, recent studies have indicated that Cec4 targets the outer membrane protein *OmpH* in *A. baumannii*, which is crucial for biofilm formation. The absence of the *OmpH* gene not only affects the thickness of the bacterial capsule but also increases the production of biofilm and makes the biofilm state of *A. baumannii* more sensitive to Cec4. This suggests that Cec4’s action on biofilms is multifaceted, involving both direct antimicrobial effects and specific interactions with bacterial proteins.

When AMPs enter the pathogen, they exert their antibacterial activity by disrupting protein folding as well as DNA and RNA synthesis. Shi et al (52). demonstrated the ability of their antimicrobial peptides to bind DNA and RNA of *Xanthomonas oryzae*, thereby hindering its migration, through techniques such as gel block detection and electrophoresis. However, the underlying mechanism remains unclear. DNA and RNA gel block tests also revealed that Cec4 is capable of binding to the genomic DNA and RNA of *K. pneumoniae*. However, a higher working concentration (256 µg/mL) is typically required to achieve noticeable inhibition of migration. To further confirm the antibacterial effect of Cec4, we constructed a CRKP wound infection model to study its efficacy *in vivo*. The results showed that treatment of the wound with Cec4 solution significantly reduced the burden of bacterial infection and promoted the proliferation of skin appendages compared with the control group. However, this effect is not due to a direct promotion of wound healing, but rather through the reduction of bacterial load. It is well known that wound healing is a complex and dynamic process that includes four main stages: coagulation, inflammation, repair, and maturation (53). In addition, the healing of wounds is closely linked to an individual’s metabolic status, especially in diabetic patients. The compromised functionality of the immune system in diabetes makes it challenging to effectively respond to infections, thereby delaying the wound healing process. Therefore, the role of Cec4 in this context should be further substantiated to provide a theoretical foundation for its clinical application.

The transcriptome data helped us to elucidate the molecular mechanism of action of Cec4. Specifically, Cec4 has the potential to disrupt the bacterial outer membrane, resulting in the compensatory upregulation of genes related to the cell membrane. For instance, *DS8_RS13340* belongs to the *YfaZ* and *VirK*/*YbjX* family of proteins and contributes to the formation of the bacterial cell membrane. Additionally, *DS8_RS26095* and the outer membrane porins *OprD* and *OprF* are essential for maintaining outer membrane functionality (54). The *bcsC* gene encodes an outer membrane protein found in the cellulose synthase complex, which regulates biofilm formation (55). Cec4 has the ability to impair the intima of *K. pneumoniae* and induce compensatory upregulation of genes associated with the cell membrane. This includes the *gspF* gene, which is ingntX, in the formligB of the intimal platform, as well as the *yiaB* gene, which has a significant relationship with intimal proteins, among others. Furthermore, consistent with the results of ROS content analysis, genes related to oxidative stress, such as the OmpK35 gene *uspF* (56), are also found to be up-regulated. These genes encode enzymes responsible for sulfhydryl peroxidase, organic hydrogen peroxide resistance, and general stress protein, all of which play a crucial role in maintaining cellular homeostasis. Finally, Cec4 treatment may also increase the metabolic demand of bacteria, mainly manifested in the up-regulation of transcription factors, ribosomal protein components, translation, and glycolysis-related genes related to DNA binding, such as *gntX*, DNA ligase *ligB*, and so on. In addition to *DNA*, *DS864_RS07825*, *DS864_RS05720*, and other genes involved in the transcription of amino acids in tRNA will be compensatively up-regulated after treatment with Cec4. In addition to up-regulated genes, the *OmpK35* gene is involved in the formation of an outer membrane barrier by outer membrane proteins in Gram-negative bacteria. The *DS864_RS15835* gene has been reported to encode a complete outer membrane protein (*Tsx*) in *E. coli*, which functions as a substrate-specific channel for deoxynucleosides and thereby affects DNA synthesis. The transcriptional regulatory factors *DS864_RS17470* (57) and *DS864_RS09470* were also significantly down-regulated after treatment with Cec4. Enrichment analysis of the aforementioned differentially expressed genes (DEGs) was conducted using the KEGG database. The results revealed significant enrichment in 11 pathways, namely the bacterial secretory system, transport system, ABC transport system, phosphate metabolism, hypophosphite metabolism, secretory system iron carrier group, non-ribosomal peptide biosynthesis, and phenylalanine metabolism. Consequently, it can be inferred that Cec4 has the potential to disrupt the normal physiological activities of bacteria by affecting the function of inner and outer membrane proteins, as well as transcriptional regulation of DNA and RNA.

### Conclusion

Cec4 exhibits antibacterial activity against CRKP *in vitro*, as it not only shows rapid bactericidal action, strong ability to resist biofilm formation, and a low likelihood of developing resistance but also disrupts cell membrane and cell wall structures, changes membrane permeability, triggers ROS accumulation, and inhibits bacterial infection by affecting DNA and RNA synthesis within the infected organism. Additionally, Cec4 can suppress CRKP infection and accelerate wound healing in a mouse model of *in vivo* infection. This study elucidates the antibacterial activity and mechanisms of action of the antimicrobial peptide Cec4 against CRKP, providing new opportunities for clinical treatment of bacterial infections.

## Data Availability

The sequencing data in the article have been deposited at the National Center for Biotechnology Information under BioProject PRJNA867035. The data used to support the findings of this study are available from the corresponding author upon request.

## SUPPLEMENTAL MATERIAL

Supplemental material spectrum.02738-24-s0001.docx Fig. S1; Tables S1 to S4.

ASM does not own the copyrights to Supplemental Material that may be linked to, or accessed through, an article. The authors have granted ASM a non-exclusive, world-wide license to publish the Supplemental Material files. Please contact the corresponding author directly for reuse.

## References

[1] Antimicrobial Resistance Collaborators. 2022. Global burden of bacterial antimicrobial resistance in 2019: a systematic analysis. Lancet 399:629–655. doi:10.1016/s0140-6736(21)02724-0 35065702 PMC8841637

[2] Musicha P, Cornick JE, Bar-Zeev N, French N, Masesa C, Denis B, Kennedy N, Mallewa J, Gordon MA, Msefula CL, Heyderman RS, Everett DB, Feasey NA. 2017. Trends in antimicrobial resistance in bloodstream infection isolates at a large urban hospital in Malawi (1998-2016): a surveillance study. Lancet Infect Dis 17:1042–1052. doi:10.1016/S1473-3099(17)30394-8 28818544 PMC5610140

[3] Rice LB. 2008. Federal funding for the study of antimicrobial resistance in nosocomial pathogens: no ESKAPE. J Infect Dis 197:1079–1081. doi:10.1086/533452 18419525

[4] Tacconelli E, Carrara E, Savoldi A, Harbarth S, Mendelson M, Monnet DL, Pulcini C, Kahlmeter G, Kluytmans J, Carmeli Y, Ouellette M, Outterson K, Patel J, Cavaleri M, Cox EM, Houchens CR, Grayson ML, Hansen P, Singh N, Theuretzbacher U, Magrini N, WHO Pathogens Priority List Working Group. 2018. Discovery, research, and development of new antibiotics: the WHO priority list of antibiotic-resistant bacteria and tuberculosis. Lancet Infect Dis 18:318–327. doi:10.1016/S1473-3099(17)30753-3 29276051

[5] Papp-Wallace KM, Endimiani A, Taracila MA, Bonomo RA. 2011. Carbapenems: past, present, and future. Antimicrob Agents Chemother 55:4943–4960. doi:10.1128/AAC.00296-11 21859938 PMC3195018

[6] Zhang F, Li Z, Liu X, Luo G, Wu Y, Li C, Zhao J, Zhang Y, Hu Y, Lu B. 2023. Molecular characteristics of an NDM-4 and OXA-181 co-producing K51-ST16 carbapenem-resistant Klebsiella pneumoniae: study of its potential dissemination mediated by conjugative plasmids and insertion sequences. Antimicrob Agents Chemother 67:e0135422. doi:10.1128/aac.01354-22 36602346 PMC9872697

[7] Jin X, Chen Q, Shen F, Jiang Y, Wu X, Hua X, Fu Y, Yu Y. 2021. Resistance evolution of hypervirulent carbapenem-resistant Klebsiella pneumoniae ST11 during treatment with tigecycline and polymyxin. Emerg Microbes Infect 10:1129–1136. doi:10.1080/22221751.2021.1937327 34074225 PMC8205050

[8] Zhang R, Dong N, Huang Y, Zhou H, Xie M, Chan E-C, Hu Y, Cai J, Chen S. 2018. Evolution of tigecycline- and colistin-resistant CRKP (carbapenem-resistant Klebsiella pneumoniae) in vivo and its persistence in the GI tract. Emerg Microbes Infect 7:127. doi:10.1038/s41426-018-0129-7 29985412 PMC6037711

[9] Wagenlehner F, Lucenteforte E, Pea F, Soriano A, Tavoschi L, Steele VR, Henriksen AS, Longshaw C, Manissero D, Pecini R, Pogue JM. 2021. Systematic review on estimated rates of nephrotoxicity and neurotoxicity in patients treated with polymyxins. Clin Microbiol Infect 27:671–686. doi:10.1016/j.cmi.2020.12.009 33359542

[10] Zavascki AP, Nation RL. 2017. Nephrotoxicity of polymyxins: is there any difference between colistimethate and polymyxin B? Antimicrob Agents Chemother 61:e02319-16. doi:10.1128/AAC.02319-16 27993859 PMC5328560

[11] Xuan J, Feng W, Wang J, Wang R, Zhang B, Bo L, Chen ZS, Yang H, Sun L. 2023. Antimicrobial peptides for combating drug-resistant bacterial infections. Drug Resist Updat 68:100954. doi:10.1016/j.drup.2023.100954 36905712

[12] Peschel A, Sahl HG. 2006. The co-evolution of host cationic antimicrobial peptides and microbial resistance. Nat Rev Microbiol 4:529–536. doi:10.1038/nrmicro1441 16778838

[13] Steiner H, Hultmark D, Engström A, Bennich H, Boman HG. 1981. Sequence and specificity of two antibacterial proteins involved in insect immunity. Nature 292:246–248. doi:10.1038/292246a0 7019715

[14] Lazzaro BP, Zasloff M, Rolff J. 2020. Antimicrobial peptides: application informed by evolution. Science 368:6490. doi:10.1126/science.aau5480 PMC8097767 32355003

[15] Nawrot R, Barylski J, Nowicki G, Broniarczyk J, Buchwald W, Goździcka-Józefiak A. 2014. Plant antimicrobial peptides. Folia Microbiol (Praha) 59:181–196. doi:10.1007/s12223-013-0280-4 24092498 PMC3971460

[16] Van Moll L, De Smet J, Paas A, Tegtmeier D, Vilcinskas A, Cos P, Van Campenhout L. 2022. In vitro evaluation of antimicrobial peptides from the black soldier fly (Hermetia Illucens) against a selection of human pathogens. Microbiol Spectr 10:e0166421. doi:10.1128/spectrum.01664-21 34985302 PMC8729770

[17] Nagarajan D, Roy N, Kulkarni O, Nanajkar N, Datey A, Ravichandran S, Thakur C, T S, Aprameya IV, Sarma SP, Chakravortty D, Chandra N. 2019. Ω76: a designed antimicrobial peptide to combat carbapenem- and tigecycline-resistant Acinetobacter baumannii. Sci Adv 5:eaax1946. doi:10.1126/sciadv.aax1946 31355341 PMC6656545

[18] Peng J, Long H, Liu W, Wu Z, Wang T, Zeng Z, Guo G, Wu J. 2019. Antibacterial mechanism of peptide Cec4 against Acinetobacter baumannii. Infect Drug Resist 12:2417–2428. doi:10.2147/IDR.S214057 31496754 PMC6689099

[19] Peng J, Mishra B, Khader R, Felix L, Mylonakis E. 2021. Novel cecropin-4 derived peptides against methicillin-resistant Staphylococcus aureus. Antibiotics (Basel) 10:36. doi:10.3390/antibiotics10010036 33401476 PMC7824259

[20] Wiegand I, Hilpert K, Hancock REW. 2008. Agar and broth dilution methods to determine the minimal inhibitory concentration (MIC) of antimicrobial substances. Nat Protoc 3:163–175. doi:10.1038/nprot.2007.521 18274517

[21] Humphries R, Bobenchik AM, Hindler JA, Schuetz AN. 2021. Overview of changes to the clinical and laboratory standards institute performance standards for antimicrobial susceptibility testing, M100, 31st edition. J Clin Microbiol 59:e0021321. doi:10.1128/JCM.00213-21 34550809 PMC8601225

[22] Li Z, Mao R, Teng D, Hao Y, Chen H, Wang X, Wang X, Yang N, Wang J. 2017. Antibacterial and immunomodulatory activities of insect defensins-DLP2 and DLP4 against multidrug-resistant Staphylococcus aureus. Sci Rep 7:12124. doi:10.1038/s41598-017-10839-4 28935900 PMC5608901

[23] Raheem N, Kumar P, Lee E, Cheng JTJ, Hancock REW, Straus SK. 2020. Insights into the mechanism of action of two analogues of aurein 2.2. Biochim Biophys Acta 1862:183262. doi:10.1016/j.bbamem.2020.183262 32147356

[24] MacNair CR, Stokes JM, Carfrae LA, Fiebig-Comyn AA, Coombes BK, Mulvey MR, Brown ED. 2018. Overcoming mcr-1 mediated colistin resistance with colistin in combination with other antibiotics. Nat Commun 9:458. doi:10.1038/s41467-018-02875-z 29386620 PMC5792607

[25] Jayathilaka E, Rajapaksha DC, Nikapitiya C, De Zoysa M, Whang I. 2021. Antimicrobial and anti-biofilm peptide octominin for controlling multidrug-resistant Acinetobacter baumannii. Int J Mol Sci 22:5353. doi:10.3390/ijms22105353 34069596 PMC8161146

[26] Kim MK, Kang N, Ko SJ, Park J, Park E, Shin DW, Kim SH, Lee SA, Lee JI, Lee SH, Ha EG, Jeon SH, Park Y. 2018. Antibacterial and antibiofilm activity and mode of action of magainin 2 against drug-resistant Acinetobacter baumannii. Int J Mol Sci 19:3041. doi:10.3390/ijms19103041 30301180 PMC6213043

[27] Wu Y, Deng S, Wang X, Thunders M, Qiu J, Li Y. 2023. Discovery and mechanism of action of a novel antimicrobial peptide from an earthworm. Microbiol Spectr 11:e0320622. doi:10.1128/spectrum.03206-22 36602379 PMC9927515

[28] Li Y, Liu F, Zhang J, Liu X, Xiao P, Bai H, Chen S, Wang D, Sung SHP, Kwok RTK, Shen J, Zhu K, Tang BZ. 2021. Efficient killing of multidrug-resistant internalized bacteria by AIEgens in vivo. Adv Sci (Weinh) 8:2001750. doi:10.1002/advs.202001750 33977040 PMC8097328

[29] Qian W, Sun Z, Wang T, Yang M, Liu M, Zhang J, Li Y. 2020. Antimicrobial activity of eugenol against carbapenem-resistant Klebsiella pneumoniae and its effect on biofilms. Microb Pathog 139:103924. doi:10.1016/j.micpath.2019.103924 31837416

[30] Shi J, Chen C, Wang D, Tong Z, Wang Z, Liu Y. 2021. Amphipathic peptide antibiotics with potent activity against multidrug-resistant pathogens. Pharmaceutics 13:438. doi:10.3390/pharmaceutics13040438 33804947 PMC8063935

[31] Liang X, Yan J, Lu Y, Liu S, Chai X. 2021. The antimicrobial peptide melectin shows both antimicrobial and antitumor activity via membrane interference and DNA binding. Drug Des Devel Ther 15:1261–1273. doi:10.2147/DDDT.S288219 PMC7989573 33776423

[32] Liu Y, Jia Y, Yang K, Li R, Xiao X, Zhu K, Wang Z. 2020. Metformin restores tetracyclines susceptibility against multidrug resistant bacteria. Adv Sci (Weinh) 7:1902227. doi:10.1002/advs.201902227 32596101 PMC7312304

[33] Kim W, Zou G, Hari TPA, Wilt IK, Zhu W, Galle N, Faizi HA, Hendricks GL, Tori K, Pan W, Huang X, Steele AD, Csatary EE, Dekarske MM, Rosen JL, Ribeiro N de Q, Lee K, Port J, Fuchs BB, Vlahovska PM, Wuest WM, Gao H, Ausubel FM, Mylonakis E. 2019. A selective membrane-targeting repurposed antibiotic with activity against persistent methicillin-resistant Staphylococcus aureus. Proc Natl Acad Sci U S A 116:16529–16534. doi:10.1073/pnas.1904700116 31358625 PMC6697817

[34] Nikapitiya C, Dananjaya SHS, Chandrarathna HPSU, De Zoysa M, Whang I. 2020. Octominin: a novel synthetic anticandidal peptide derived from defense protein of Octopus minor. Mar Drugs 18:56. doi:10.3390/md18010056 31952292 PMC7024321

[35] Li T, Liu Q, Wang D, Li J. 2019. Characterization and antimicrobial mechanism of CF-14, a new antimicrobial peptide from the epidermal mucus of catfish. Fish Shellfish Immunol 92:881–888. doi:10.1016/j.fsi.2019.07.015 31291603

[36] Hou X, Li S, Luo Q, Shen G, Wu H, Li M, Liu X, Chen A, Ye M, Zhang Z. 2019. Discovery and identification of antimicrobial peptides in Sichuan pepper (Zanthoxylum bungeanum Maxim) seeds by peptidomics and bioinformatics. Appl Microbiol Biotechnol 103:2217–2228. doi:10.1007/s00253-018-09593-y 30623204

[37] Hou X, Li J, Tang H, Li Q, Shen G, Li S, Chen A, Peng Z, Zhang Y, Li C, Zhang Z. 2022. Antibacterial peptide NP-6 affects Staphylococcus aureus by multiple modes of action. Int J Mol Sci 23:7812. doi:10.3390/ijms23147812 35887160 PMC9319634

[38] Guo K, Zhang M, Cai J, Ma Z, Fang Z, Zhou H, Chen J, Gao M, Wang L. 2022. Peptide‐engineered AIE nanofibers with excellent and precisely adjustable antibacterial activity. Small 18:e2108030. doi:10.1002/smll.202108030 35307954

[39] Ge B, Wang H, Li J, Liu H, Yin Y, Zhang N, Qin S. 2020. Comprehensive assessment of Nile tilapia skin (Oreochromis niloticus) collagen hydrogels for wound dressings. Mar Drugs 18:178. doi:10.3390/md18040178 32218368 PMC7230254

[40] Curren EJ, Lutgring JD, Kabbani S, Diekema DJ, Gitterman S, Lautenbach E, Morgan DJ, Rock C, Salerno RM, McDonald LC. 2022. Advancing diagnostic stewardship for healthcare-associated infections, antibiotic resistance, and sepsis. Clin Infect Dis 74:723–728. doi:10.1093/cid/ciab672 34346494

[41] Wang M, Earley M, Chen L, Hanson BM, Yu Y, Liu Z, Salcedo S, Cober E, Li L, Kanj SS, et al.. 2022. Clinical outcomes and bacterial characteristics of carbapenem-resistant Klebsiella pneumoniae complex among patients from different global regions (CRACKLE-2): a prospective, multicentre, cohort study. Lancet Infect Dis 22:401–412. doi:10.1016/S1473-3099(21)00399-6 34767753 PMC8882129

[42] Brady D, Grapputo A, Romoli O, Sandrelli F. 2019. Insect cecropins, antimicrobial peptides with potential therapeutic applications. Int J Mol Sci 20:5862. doi:10.3390/ijms20235862 31766730 PMC6929098

[43] Shi J, Chen C, Wang D, Wang Z, Liu Y. 2022. The antimicrobial peptide LI14 combats multidrug-resistant bacterial infections. Commun Biol 5:926. doi:10.1038/s42003-022-03899-4 36071151 PMC9452538

[44] Mao C, Wang Y, Yang Y, Li L, Yuan K, Cao H, Qiu Z, Guo G, Wu J, Peng J. 2022. Cec4-derived peptide inhibits planktonic and biofilm-associated methicillin resistant Staphylococcus epidermidis. Microbiol Spectr 10:e0240922. doi:10.1128/spectrum.02409-22 36453944 PMC9769716

[45] Jepson AK, Schwarz-Linek J, Ryan L, Ryadnov MG, Poon WCK. 2016. What is the 'minimum inhibitory concentration' (MIC) of pexiganan acting on Escherichia coli?-a cautionary case study. Adv Exp Med Biol 915:33–48. doi:10.1007/978-3-319-32189-9_4 27193536

[46] Perron GG, Zasloff M, Bell G. 2006. Experimental evolution of resistance to an antimicrobial peptide. Proc R Soc B 273:251–256. doi:10.1098/rspb.2005.3301 PMC1560030 16555795

[47] Choi J, Jang A, Yoon YK, Kim Y. 2021. Development of novel peptides for the antimicrobial combination therapy against carbapenem-resistant Acinetobacter baumannii infection. Pharmaceutics 13:1800. doi:10.3390/pharmaceutics13111800 34834215 PMC8619914

[48] Yin W, Wang Y, Liu L, He J. 2019. Biofilms: the microbial “protective clothing” in extreme environments. Int J Mol Sci 20:3423. doi:10.3390/ijms20143423 31336824 PMC6679078

[49] Zhang QY, Yan ZB, Meng YM, Hong XY, Shao G, Ma JJ, Cheng XR, Liu J, Kang J, Fu CY. 2021. Antimicrobial peptides: mechanism of action, activity and clinical potential. Mil Med Res 8:48. doi:10.1186/s40779-021-00343-2 34496967 PMC8425997

[50] Strahl H, Errington J. 2017. Bacterial membranes: structure, domains, and function. Annu Rev Microbiol 71:519–538. doi:10.1146/annurev-micro-102215-095630 28697671

[51] Yu L, Fan Q, Yue X, Mao Y, Qu L. 2015. Activity of a novel-designed antimicrobial peptide and its interaction with lipids. J Pept Sci 21:274–282. doi:10.1002/psc.2728 25683050

[52] Shi W, Li C, Li M, Zong X, Han D, Chen Y. 2016. Antimicrobial peptide melittin against Xanthomonas oryzae pv. oryzae, the bacterial leaf blight pathogen in rice. Appl Microbiol Biotechnol 100:5059–5067. doi:10.1007/s00253-016-7400-4 26948237 PMC4866983

[53] Wilkinson HN, Hardman MJ. 2020. Wound healing: cellular mechanisms and pathological outcomes. Open Biol 10:200223. doi:10.1098/rsob.200223 32993416 PMC7536089

[54] Chevalier S, Bouffartigues E, Bodilis J, Maillot O, Lesouhaitier O, Feuilloley MGJ, Orange N, Dufour A, Cornelis P. 2017. Structure, function and regulation of Pseudomonas aeruginosa porins. FEMS Microbiol Rev 41:698–722. doi:10.1093/femsre/fux020 28981745

[55] Sharma N, Das A, Raja P, Marathe SA. 2022. The CRISPR-Cas system differentially regulates surface-attached and pellicle biofilm in Salmonella enterica serovar Typhimurium. Microbiol Spectr 10:e0020222. doi:10.1128/spectrum.00202-22 35678575 PMC9241790

[56] de Souza CS, Torres AG, Caravelli A, Silva A, Polatto JM, Piazza RMF. 2016. Characterization of the universal stress protein F from atypical enteropathogenic Escherichia coli and its prevalence in Enterobacteriaceae. Protein Sci 25:2142–2151. doi:10.1002/pro.3038 27616205 PMC5119564

[57] Tian Z-X, Fargier E, Mac Aogáin M, Adams C, Wang Y-P, O’Gara F. 2009. Transcriptome profiling defines a novel regulon modulated by the LysR-type transcriptional regulator MexT in Pseudomonas aeruginosa. Nucleic Acids Res 37:7546–7559. doi:10.1093/nar/gkp828 19846594 PMC2794183

