# Supplementary material for: The antimicrobial peptide Cec4 has therapeutic potential against clinical carbapenem-resistant Klebsiella pneumoniae
Source: Microbiol Spectr. 2025 May 16;13(7):e02738-24. doi: 10.1128/spectrum.02738-24 (PMC12210888; doi:10.1128/spectrum.02738-24)
Supplement: Supplemental material — Fig. S1; Tables S1 to S4. [file spectrum.02738-24-s0001.docx]

Supplementary Material

1. **Experimental procedure**

**1.1 Molecular dynamics simulations**

The MD simulations were conducted based on previous studies with minor modifications[1]. Briefly, CHARMM-GUI was utilized to construct a phospholipid bilayer consisting of 256 phospholipids, with a ratio of 3:1 for palmitoyloleoylphosphatidylglycerol (POPG) and palmitoyloleoylphosphatidylethanolamine (POPE). The structure of Cec4 was predicted by AlphFold2.

The MD simulations and analyses were performed using GROMACS 2022 (https://github.com/gromacs/gromacs) and the CHARMM36m force field. 100 ns MD simulations of Cec4 were performed in the presence of solution and membranes, in which water molecules were described by the TIP3P model and neutralized with K^+^ and Cl^−^ counterions. The temperatures for the simulations were kept at 300 K. The molecular mechanics Poisson Boltzmann surface area (MM-PBSA) method and the decomposition scheme in gmx_MMPBSA 1.6.1 (gmx_MMPBSA v1.6.1 based on MMPBSA version 16.0 and AmberTools 20) were utilized to calculate the binding free energy (DGbind). The relative binding free energy was used, and the entropy contribution of the peptide was neglected.

**1.2 Bacteria Culturing, Staining, and Imaging**

The distribution of FITC-Cec4 in *K. pneumoniae* was observed by CLSM [2]. Bacteria (1 × 10^8^ CFU/mL) were incubated with FITC-Cec4 (8 μg/mL) for 1.5 h. After three washes with PBS, 4′,6-diamidino-2-phenylindole (DAPI, 2 μg/mL) and N-(3-Triethylammoniumpropyl)-4-(6-(4-(Diethylamino) Phenyl) Hexatrienyl) Pyridinium Dibromide (FM-4-64, 10 μg/mL) were co-cultured at 37 °C for 5 min.

The bacterial suspension was prepared in the same way, and different concentrations of Cec4 peptide solution (0–16 μg/mL) were added for 1.5 h. Subsequently, PI and SYTO 9 (5 μM; Invitrogen, USA) were added and incubated in darkness for 15 min in CLSM (Olympus, FV1000, Japan) for observation and recording.

According to the reference method, bacterial death was observed using a flow cytometer [3]. Bacterial suspensions (10^6^ CFUs/mL) were incubated with Cec4 peptide (0–16 μg/mL) for 1.5 h at 37 °C. Then, PI (10 μM) was added to the mixture of bacteria and Cec4 peptide and incubated for 15 min at room temperature in the dark. Approximately 100,000 ungated events were measured with a CytExpert Flow Cytometer (Beckman, USA) and analyzed with CytExpert 2.0 software (Beckman, USA) (λexcitation = 488 nm, λemission = 495 nm).


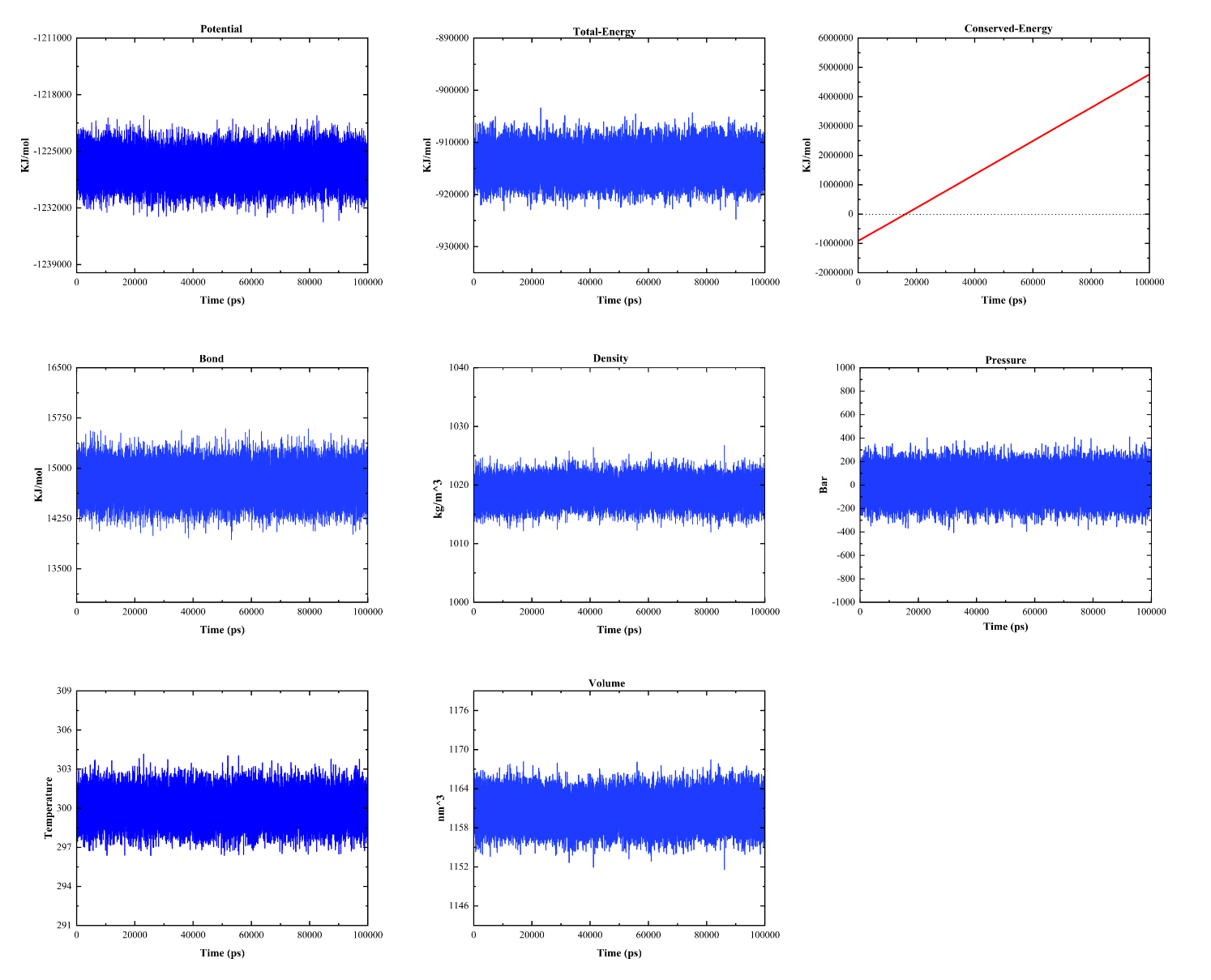


**Figure S1.** System stability for molecular dynamics simulations. The Potential, Total-Energy, Conserved-Energy, Bond, Density, Pressure, Temperature and Volume in the system are relatively stable during the MD simulation within 0 ns to 100 ns.

**Table S1. Physicochemical Parameters of Cec4**

| Peptide | Sequence (N→C) | Formula | Charge^a^ | pI^a^ | Calcd  MW^a^ | Obsd  MW^b^ |
| --- | --- | --- | --- | --- | --- | --- |
| Cec4 | GWLKKIG KKIERVGQNTRDATIQAIGVAQQAANVAAT LKGK | C_190_H_328_N_60_O_55_ | +6 | 10.66 | 4333.06 | 4332.02 |

Note: ^a^Charge, isoelectric point (pI) and molecular weight (MW) were calculated online at https://web.expasy.org/protparam/. ^b^The observed molecular weight (MW) was determined by liquid chromatograph mass spectrometer (LC-MS).

**Table S2. Strain information and MIC value of *Klebsiella pneumoniae***

| Strain | Origin | MIC(μg/mL) | | |
| --- | --- | --- | --- | --- |
|  |  | Cec4 | Imipenem | Meropenem |
| CRKP32 | Sputum | 8 | 512 | 256 |
| CRKP33 | Ascites | 8 | 256 | 256 |
| CRKP34 | Sputum | 8 | 512 | 256 |
| CRKP37 | Sputum | 8 | 512 | 256 |
| CRKP41 | Sputum | 8 | 128 | 512 |
| CRKP45 | Sputum | 8 | 64 | 256 |
| CRKP46 | Sputum | 8 | 256 | 256 |
| CRKP61 | Sputum | 8 | 512 | 512 |
| CRKP63 | Blood | 8 | 256 | 256 |
| CRKP64 | Sputum | 8 | 256 | 256 |
| CRKP85 | Sputum | 8 | 64 | 128 |
| CRKP86 | Sputum | 8 | 64 | 512 |
| CRKP94 | Sputum | 8 | 32 | 256 |
| CRKP95 | Sputum | 8 | 32 | 256 |
| CRKP96 | Blood | 8 | 32 | 256 |
| CRKP97 | Central Catheter | 8 | 32 | 512 |
| CRKP99 | Sputum | 8 | 256 | 512 |
| CRKP100 | Sputum | 8 | 64 | 64 |
| CRKP101 | Sputum | 8 | 32 | 16 |
| CRKP104 | Blood | 8 | 128 | 256 |
| CRKP105 | Urine | 8 | 128 | 128 |
| CRKP109 | Sputum | 8 | 128 | 256 |
| CRKP111 | Sputum | 8 | 256 | 256 |
| CRKP112 | Sputum | 8 | 128 | 256 |
| CRKP119 | Sputum | 8 | 128 | 512 |
| CRKP125 | Sputum | 8 | 256 | 64 |
| CRKP153 | Blood | 8 | 32 | 32 |
| CRKP157 | Blood | 8 | 256 | 512 |
| CRKP158 | Sputum | 8 | 128 | 256 |
| CRKP159 | Ascites | 8 | 64 | 128 |
| CRKP163 | Secretion | 8 | 128 | 256 |
| CRKP164 | Excrement | 8 | 64 | 128 |
| CRKP166 | Sputum | 8 | >512 | 512 |
| CRKP167 | Sputum | 8 | 512 | 256 |
| CRKP168 | Urine | 8 | 256 | 256 |
| CRKP171 | Blood | 8 | 64 | 64 |
| CRKP173 | Urine | 8 | 64 | 64 |
| CRKP177 | Urine | 8 | 64 | 64 |
| CRKP178 | Sputum | 8 | 64 | 64 |
| CRKP180 | Sputum | 8 | 128 | 128 |
| CRKP182 | Sputum | 8 | 64 | 64 |
| CRKP195 | Urine | 8 | 64 | 64 |
| CRKP197 | Excrement | 8 | 32 | 32 |
| CRKP198 | Cerebrospinal Fluid | 8 | 32 | 32 |
| CRKP200 | Urine | 8 | 64 | 64 |
| CRKP203 | Sputum | 8 | 64 | 64 |
| CRKP205 | Urine | 8 | 64 | 64 |
| CRKP206 | Sputum | 4 | 64 | 128 |

**Supplementary Table S3. Genes with significantly altered transcript levels following Cec4 treatment**

| **Gene name** | **Description** | **log2 Fold change** | **Corrected *P* value** |
| --- | --- | --- | --- |
| **Outer membrane** | |  |  |
| DS864_RS26410 | fimbrial biogenesis outer membrane usher protein | 6.06 | 9.01×10^-6^ |
| DS864_RS17985 | fimbrial biogenesis outer membrane usher protein | 5.42 | 3.78×10^-15^ |
| DS864_RS15485 | fimbrial biogenesis outer membrane usher protein | 5.37 | 6.11×10^-12^ |
| DS864_RS26610 | MdtP family multidrug efflux transporter outer membrane subunit | 4.71 | 2.13×10^-12^ |
| DS864_RS13340 | YfaZ family outer membrane protein | 4.53 | 4.27×10^-20^ |
| DS864_RS00660 | fimbrial biogenesis outer membrane usher protein | 4.52 | 1.91×10^-6^ |
| DS864_RS26095 | OprD family outer membrane porin | 4.51 | 4.10×10^-10^ |
| DS864_RS08555 | fimbrial biogenesis outer membrane usher protein | 4.32 | 7.34×10^-5^ |
| bcsC | cellulose synthase complex outer membrane protein BcsC | 4.03 | 1.04×10^-11^ |
| ompK35 | porin OmpK35 | -6.29 | 1.93×10^-37^ |
| **Inner membrane** | |  |  |
| DS864_RS07850 | Binding-protein-dependent transport system inner membrane component | 7.93 | 6.48×10^-10^ |
| phnE | Binding-protein-dependent transport system inner membrane component | 7.20 | 6.30×10^-7^ |
| DS864_RS00195 | Binding-protein-dependent transport system inner membrane component | 7.02 | 2.16×10^-10^ |
| DS864_RS21610 | Binding-protein-dependent transport system inner membrane component | 7.00 | 6.84×10^-10^ |
| DS864_RS00390 | Binding-protein-dependent transport system inner membrane component | 6.94 | 5.15×10^-8^ |
| DS864_RS07845 | Binding-protein-dependent transport system inner membrane component | 6.84 | 5.74×10^-6^ |
| DS864_RS21615 | ABC transporter permease | 6.63 | 6.16×10^-12^ |
| DS864_RS22725 | ABC transporter permease subunit | 6.55 | 3.91×10^-12^ |
| DS864_RS06315 | ABC transporter permease | 6.28 | 4.35×10^-15^ |
| DS864_RS17120 | ABC transporter permease | 5.81 | 2.12×10^-9^ |
| DS864_RS15500 | amino acid ABC transporter permease | 5.78 | 1.55×10^-13^ |
| gspF | type II secretion system inner membrane protein GspF | 5.77 | 4.57×10^-16^ |
| DS864_RS00385 | amino acid ABC transporter permease | 5.65 | 5.81×10^-8^ |
| DS864_RS02720 | ABC transporter permease subunit | 5.64 | 6.27×10^-19^ |
| DS864_RS01815 | amino acid ABC transporter permease/ATP-binding protein | 5.58 | 9.15×10^-9^ |
| DS864_RS15505 | amino acid ABC transporter permease | 4.90 | 1.77×10^-10^ |
| yiaB | inner membrane protein YiaB | 4.87 | 5.59×10^-3^ |
| DS864_RS06310 | ABC transporter permease | 4.79 | 1.32×10^-8^ |
| DS864_RS20495 | ABC transporter permease | 4.79 | 1.57×10^-10^ |
| ntrB | nitrate ABC transporter permease | 4.77 | 8.39×10^-7^ |
| phnV | 2-aminoethylphosphonate ABC transport system%2C membrane component PhnV | 4.72 | 3.57×10^-8^ |
| DS864_RS00100 | Putative inner membrane exporter, YdcZ | 4.71 | 9.41×10^-6^ |
| nikC | nickel ABC transporter permease subunit NikC | 4.57 | 1.09×10^-11^ |
| DS864_RS21840 | amino acid ABC transporter permease | 4.54 | 6.24×10^-7^ |
| phnE | phosphonate ABC transporter%2C permease protein PhnE | 4.52 | 3.21×10^-8^ |
| DS864_RS22580 | iron ABC transporter permease | 4.26 | 8.11×10^-11^ |
| cadB | cadaverine/lysine antiporter | 4.24 | 1.24×10^-14^ |
| **Electron Transport** | |  |  |
| ybtU | yersiniabactin biosynthesis oxidoreductase YbtU | 6.33 | 2.58×10^-6^ |
| DS864_RS17105 | FAD/NAD(P)-binding protein | 6.24 | 1.03×10^-18^ |
| DS864_RS06340 | Gfo/Idh/MocA family oxidoreductase | 6.05 | 1.27×10^-14^ |
| DS864_RS16820 | SLBB domain-containing protein | 5.06 | 2.51×10^-21^ |
| norW | NADH:flavorubredoxin reductase NorW | 4.80 | 7.53×10^-12^ |
| DS864_RS06015 | 2-hydroxyacid dehydrogenase | 4.77 | 2.59×10^-12^ |
| DS864_RS01800 | NAD(P)/FAD-dependent oxidoreductase | 4.61 | 7.93×10^-8^ |
| DS864_RS26080 | aspartate dehydrogenase | 4.51 | 5.18×10^-7^ |
| DS864_RS02325 | SDR family oxidoreductase | 4.42 | 6.27×10^-11^ |
| DS864_RS22780 | acetaldehyde dehydrogenase (acetylating) | 4.39 | 7.87×10^-7^ |
| DS864_RS06540 | Gfo/Idh/MocA family oxidoreductase | 4.16 | 5.07×10^-9^ |
| benC | benzoate 1%2C2-dioxygenase electron transfer component BenC | 4.07 | 9.52×10^-5^ |
| ligB | NAD-dependent DNA ligase LigB | 4.01 | 1.14×10^-10^ |
| **Amino acid transport** | |  |  |
| DS864_RS06000 | ABC transporter permease | 7.05 | 8.13×10^-14^ |
| urtB | urea ABC transporter permease subunit UrtB | 6.46 | 4.93×10^-15^ |
| DS864_RS06005 | ABC transporter permease | 6.09 | 3.98×10^-12^ |
| DS864_RS15535 | branched-chain amino acid ABC transporter permease | 5.56 | 5.61×10^-10^ |
| DS864_RS10310 | ABC transporter permease | 4.86 | 1.05×10^-8^ |
| gguB | sugar ABC transporter permease | 4.41 | 9.63×10^-12^ |
| DS864_RS06085 | ABC transporter permease | 4.35 | 6.93×10^-9^ |
| DS864_RS08170 | ABC transporter permease | 4.34 | 5.99×10^-11^ |
| urtC | urea ABC transporter permease subunit UrtC | 4.10 | 3.78×10^-7^ |
| **Stress protein** |  |  |  |
| yhcN | peroxide/acid stress response protein YhcN | 4.69 | 2.02×10^-30^ |
| yhcN-B | DUF1471 family stress response protein YhcN-B | 4.13 | 9.55×10^-21^ |
| uspF | universal stress protein UspF | 4.07 | 9.35×10^-5^ |
| pspG | envelope stress response protein PspG | 4.06 | 4.81×10^-18^ |
| **RNA** |  |  |  |
| DS864_RS07825 | tRNA-Leu | 8.67 | 8.78×10^-9^ |
| DS864_RS05720 | tRNA-Leu | 7.12 | 3.83×10^-7^ |
| DS864_RS05735 | tRNA-Met | 6.24 | 2.54×10^-5^ |
| DS864_RS17790 | tRNA-Arg | 5.60 | 3.33×10^-10^ |
| DS864_RS17780 | tRNA-Arg | 4.77 | 2.46×10^-20^ |
| DS864_RS17785 | tRNA-Arg | 4.74 | 1.47×10^-12^ |
| DS864_RS05740 | tRNA-Gln | 4.34 | 5.67×10^-7^ |
| DS864_RS05380 | tRNA-Lys | 4.30 | 3.23×10^-3^ |
| **DNA** |  |  |  |
| DS864_RS00165 | ATP-binding protein | 5.27 | 4.03×10^-13^ |
| DS864_RS20565 | sensor histidine kinase | 5.24 | 1.63×10^-12^ |
| DS864_RS22805 | DNA-binding transcriptional regulator | 5.12 | 8.33×10^-10^ |
| gntX | DNA utilization protein GntX | 4.85 | 3.20×10^-13^ |
| DS864_RS06345 | LacI family DNA-binding transcriptional regulator | 4.12 | 2.35×10^-9^ |
| baeS | two-component system sensor histidine kinase BaeS | 4.07 | 5.31×10^-11^ |
| ligB | NAD-dependent DNA ligase LigB | 4.01 | 1.14×10^-10^ |
| DS864_RS15835 | nucleoside-specific channel-forming protein Tsx | -4.03 | 2.32×10^-16^ |
| **Others** |  |  |  |
| DS864_RS17470 | LysR family transcriptional regulator | -4.12 | 1.19×10^-25^ |
| DS864_RS09470 | anaerobic C4-dicarboxylate transporter | -4.21 | 1.33×10^-25^ |
| DS864_RS20475 | HAD-IA family hydrolase | -4.49 | 1.83×10^-13^ |
| DS864_RS09470 | complement resistance protein TraT | -4.52 | 1.25×10^-30^ |
| DS864_RS20460 | mannitol dehydrogenase family protein | -5.36 | 3.22×10^-42^ |
| DS864_RS09180 | maltoporin | -5.52 | 6.06×10^-29^ |
| DS864_RS06650 | cation-transporting P-type ATPase | 4.05 | 1.33×10^-15^ |
| dalT | D-arabinitol transporter | -6.25 | 4.06×10^-44^ |

**Supplementary Table S4** Primers used for RNA-seq was validated by qRT-PCR.

| Target gene | The sequence of primers (5’ to 3’) | | Amplicon (bp) |
| --- | --- | --- | --- |
| DS864_RS13340 | F | TCAATCAGGGTGTGGTTCGG | 120 |
|  | R | CTATGTTGAAGCGAACGGCG |  |
| ompK35 | F | GACGATACCACCTATGCCCG | 141 |
|  | R | GAACGCCAGACGGGTTTTTG |  |
| DS864_RS27220 | F | AGAGCGGCAACATTATCCGT | 124 |
|  | R | CTGCTGGTCTGGTGGGTATG |  |
| arnB | F | GGACGCTTATGATCGCCAGA | 120 |
|  | R | CGTGGCTCAGTTTTTCCAGC |  |
| DS864_RS06650 | F | CAGGATAACGGCGAAGTGGT | 113 |
|  | R | TTGGTGACTTCGGTGCCTTT |  |
| DS864_RS15835 | F | GTTGCGGCCCATATCGTAGA | 130 |
|  | R | AGGTTCCCCGCTGTTTATGG |  |
| yhcN-B | F | TTTAGCCGCCAGTTCCTGAC | 110 |
|  | R | TCCAGCAAGTGAACGCAGAG |  |
| DS864_RS09470 | F | GGGCAGGATAAAGCTGTGGT | 95 |
|  | R | CGACCTACCCAAGCGATCTG |  |
| rpoB | F | GGTAATTCCGAGCTGCAATACG | 133 |
|  | R | CGCGCTCGTAGATCACCAG |  |

**References**

[1] Y. Wu, S. Deng, X. Wang, M. Thunders, J. Qiu, Y. Li, Discovery and Mechanism of Action of a Novel Antimicrobial Peptide from an Earthworm, Microbiology spectrum 11(1) (2023) e0320622.

[2] R. Zhang, Z. Wang, Y. Tian, Q. Yin, X. Cheng, M. Lian, B. Zhou, X. Zhang, L. Yang, Efficacy of Antimicrobial Peptide DP7, Designed by Machine-Learning Method, Against Methicillin-Resistant Staphylococcus aureus, Frontiers in microbiology 10 (2019) 1175.

[3] Z. Li, R. Mao, D. Teng, Y. Hao, H. Chen, X. Wang, X. Wang, N. Yang, J. Wang, Antibacterial and immunomodulatory activities of insect defensins-DLP2 and DLP4 against multidrug-resistant Staphylococcus aureus, Scientific reports 7(1) (2017) 12124.
